# Valproic Acid Teratogenicity: A Toxicogenomics Approach

**DOI:** 10.1289/txg.7034

**Published:** 2004-06-03

**Authors:** Kim Kultima, Anna-Maja Nyström, Birger Scholz, Anne-Lee Gustafson, Lennart Dencker, Michael Stigson

**Affiliations:** Department of Pharmaceutical Biosciences, Division of Toxicology, The Biomedical Center, Uppsala University, Uppsala, Sweden

**Keywords:** biomarker, embryocarcinoma, galectin-1, histone deacetylase, *in vitro* toxicology, metallothionein, microarray, mouse embryo, neural tube defect, Sp1, teratogen, valproic acid, vinculin

## Abstract

Embryonic development is a highly coordinated set of processes that depend on hierarchies of signaling and gene regulatory networks, and the disruption of such networks may underlie many cases of chemically induced birth defects. The antiepileptic drug valproic acid (VPA) is a potent inducer of neural tube defects (NTDs) in human and mouse embryos. As with many other developmental toxicants however, the mechanism of VPA teratogenicity is unknown. Using microarray analysis, we compared the global gene expression responses to VPA in mouse embryos during the critical stages of teratogen action *in vivo* with those in cultured P19 embryocarcinoma cells *in vitro*. Among the identified VPA-responsive genes, some have been associated previously with NTDs or VPA effects [vinculin, metallothioneins 1 and 2 (*Mt1*, *Mt2*), keratin 1-18 (*Krt1-18*)], whereas others provide novel putative VPA targets, some of which are associated with processes relevant to neural tube formation and closure [transgelin 2 (*Tagln2*), thyroid hormone receptor interacting protein 6, galectin-1 (*Lgals1*), inhibitor of DNA binding 1 (*Idb1*), fatty acid synthase (*Fasn*), annexins A5 and A11 (*Anxa5*, *Anxa11*)], or with VPA effects or known molecular actions of VPA (*Lgals1*, *Mt1*, *Mt2*, *Id1*, *Fasn*, *Anxa5*, *Anxa11*, *Krt1-18*). A subset of genes with a transcriptional response to VPA that is similar in embryos and the cell model can be evaluated as potential biomarkers for VPA-induced teratogenicity that could be exploited directly in P19 cell–based *in vitro* assays. As several of the identified genes may be activated or repressed through a pathway of histone deacetylase (HDAC) inhibition and specificity protein 1 activation, our data support a role of HDAC as an important molecular target of VPA action *in vivo*.

Exposure during pregnancy to pharmaceuticals and environmental chemicals remains a worldwide problem. Assessing risk for human developmental toxicity is a major obstacle in drug development, as it relies on data from animal experiments, with associated concordance problems. A common understanding of basal mechanisms of developmental toxicity could assist risk assessment, but such mechanisms have unfortunately remained elusive. How individual teratogenic agents induce early developmental errors, and how widely different teratogens induce apparently similar defects by common or distinct mechanisms are still largely unknown. Compared with most established adult organs, the mammalian embryo comprises a moving target of highly dynamic cell interactions. This inherent complexity impedes the mechanistic interpretation of a chemical insult and may ultimately preclude what appear as more desirable *in vitro* methods from completely replacing whole-animal experiments in developmental toxicology. Nevertheless, cell-based screening methods could be devised based on knowledge of molecular mechanisms, pathways, and biomarkers of toxicity.

Recently, toxicogenomics has emerged as an attractive approach to uncover critical molecular events altered by toxicants (Aardema and MacGregor 2002; Iannaccone 2001; Nuwaysir et al. 1999). Using microarrays and profiling techniques, investigators can determine how gene expression responses to toxic exposure are linked to toxic outcome (phenotypic anchoring) (Paules 2003) and identify molecular targets and biomarkers of chemically induced toxicity. However, few microarray studies so far have addressed developmental toxicity (Docterman and Smith 2002) or embryonic development (Ko 2001; Smith and Greenfield 2003). We predict that disruption of the hierarchies of signaling and gene regulatory networks that control embryonic development may underlie many cases of chemically induced birth defects. Teratogenic chemicals are therefore likely to affect downstream gene expression as a cause or consequence, or both, of their adverse developmental effects. Hence, compound-specific gene expression responses should be possible to detect.

In this study we used spotted cDNA microarrays to monitor global gene expression changes in response to the antiepileptic drug valproic acid (VPA), a potent teratogen that most notably induces neural tube defects (NTDs) in human, mouse, and other vertebrate embryos (Lammer et al. 1987; Nau et al. 1991; Oberemm and Kirschbaum 1992; Whitsel et al. 2002). NTDs with varying penetrance can be induced in the mouse embryo by many chemical treatments (Copp et al. 1990) and by the functional disruption of a plethora of genes (Copp et al. 2003; Juriloff and Harris 2000). Induction and development of NTDs in the mouse embryo is thus a relevant model for studying chemically induced teratogenicity. In this context, we believe that VPA is a good model substance to be addressed by a toxicogenomics approach. Although the molecular mechanism by which VPA causes NTDs remains obscure, several genes and molecular targets have been associated with VPA action, both in embryos (Craig et al. 2000; Faiella et al. 2000; Wlodarczyk et al. 1996) and various cell lines (Blaheta and Cinatl 2002; Phiel et al. 2001; Walmod et al. 1999; Werling et al. 2001; Yuan et al. 2001) and therapeutically in epilepsy and bipolar disorders (Gurvich and Klein 2002; Johannessen 2000). We report here the altered expression of multiple genes in mouse embryos after treatment with VPA, and discuss some of these genes in the light of neural tube development and previously known VPA actions. Employing the mouse embryocarcinoma cell line P19 as an *in vitro* model of early pluripotent embryonic cells, we identify further a subset of VPA-responsive genes that may be particularly relevant to evaluate as potential biomarkers of VPA teratogenicity.

## Materials and Methods

### Embryos

NMRI mice (B&K Universal AB, Sollentuna, Sweden) were kept on a 12-hr light cycle (1100–2200 hr) in the Laboratory Animal Facility at The Biomedical Center. Females were mated with males for 2 hr at the end of the dark period (0800–1000 hr). Females were then checked for vaginal plugs, and the midpoint of the mating period (0900 hr) was taken as 0 days postcoitum (dpc) Pregnant dams were treated 8.0 dpc by ip injection of 600 mg/kg body weight sodium valproate (Sigma Chemical Co., St. Louis, MO, USA) in approximately 100–200 μL 0.9% saline; control mice received saline only. Dams were sacrificed by cervical dislocation at 1.5 hr [RNA for quantitative reverse transcription-polymerase chain reaction (RT-PCR)], 6 hr (RNA for microarrays and quantitative RT-PCR), or 48 hr [morphological examination and detection of programmed cell death (PCD) by terminal deoxynucleotidyl transferase-mediated (dUTP) biotinylated nick end labeling (TUNEL) staining] posttreatment. The uterus was quickly transferred to phosphate-buffered saline (PBS), pH 7.4, and the embryos were removed. For RNA preparation, embryos were lysed in Trizol reagent (Invitrogen, Carlsbad, CA, USA), and stored at −80°C until further use. Because of some within-litter and between-litter variation in the size and developmental stage of 8.25-dpc embryos (6 hr posttreatment), each embryo was quickly evaluated morphologically before lysis, and embryos that appeared younger than the late bud stage, as defined by Downs and Davies (1993) were excluded. Three pools each of treated and control embryos were created containing embryos from 2, 3, and 12 VPA-treated litters or 3, 4, and 12 control litters, respectively.

### Detection of Programmed Cell Death

Embryos removed from control and treated animals were fixed overnight in 4% paraformaldehyde (PF) in PBT (0.1% Tween-20 in PBS), then processed into 100% methanol and stored at −20°C until use. PCD detection was performed using the *In Situ* Cell Death Detection Kit, AP (Roche Diagnostics, Indianapolis, IN, USA) according to manufacturer instructions, with the following minor changes: permeabilization of the embryos was performed for 10 min in 10 μg/mL proteinase K, followed by 4% PF for 10 min, before inactivation of the endogenous peroxidase. All washing steps were performed with PBT.

### Cell Culture

P19 mouse embryocarcinoma cells (ATCC CRL-1825; American Type Culture Collection, Manassas, VA, USA) were cultured at 37°C and 5% CO_2_ in Dulbecco’s modified Eagle’s medium (National Veterinary Institute, Uppsala, Sweden) supplemented with 10% fetal bovine serum (Seromed, Berlin, Germany), 1% l-glutamine, and 1% penicillin/streptavidin. Cells from a subconfluent T75 flask were split 1:20 onto 10-cm plates (Nunc, Roskilde, Denmark) in 10 mL medium; the next day half the plates were treated with 1 μM sodium valproate by adding 10 μL from a 1-mM stock solution; control plates received 10 μL water. After incubation for 24 hr, the plates were washed twice with PBS, and the cells were lysed with 3 mL Trizol reagent per plate for 5 min at room temperature. Genomic DNA was sheared by drawing the lysate several times through a pipette until it appeared nonviscous. Subsequently, the lysates from all 10 treated and control plates were pooled and stored at −80°C until further use.

### RNA Isolation

Total RNA was isolated from frozen embryos and cells using Trizol reagent (Invitrogen) according to manufacturer instructions. RNA concentration was determined spectrophotometrically, and RNA quality was checked using the Agilent 2100 Bioanalyzer and the RNA 6000 LabChip kit (Agilent Technologies, Palo Alto, CA, USA). The yield was approximately 1 μg per 8.25-dpc embryo and 300 μg per confluent plate of P19 cells. The A260/A280 ratios ranged from 1.8–2.0, and the 28S/18S ribosomal RNA ratios were approximately 1.8.

### cDNA Synthesis

Equal amounts of RNA from control and treated samples were separately converted to fluorescently labeled cDNA by incorporation of the dye-conjugated nucleotides Cyanine 3 (Cy3)-dCTP or Cy5-dCTP (or vice versa) during first-strand cDNA synthesis. Briefly, 30 μg embryonic RNA or 50 μg cellular RNA was mixed with anchored dT_17_ primer, heated to 70°C and reverse transcribed with Superscript II reverse transcriptase (Invitrogen) for 2 hr at 42°C in the presence of 100 μM fluorescent nucleotide. After hydrolysis of the RNA template, unincorporated fluorescent nucleotides were removed by ethanol precipitation.

### Microarray Hybridization

Spotted cDNA microarrays [mouse NIA clone set arrays, slide 1 (http://www.hgmp.mrc.ac.uk/Research/Microarray/HGMP-RC_Microarrays/array_description_files.jsp)], containing a subset of 6,144 clones from approximately 15,000 developmentally expressed mouse genes in the National Institute on Aging (NIA) 15 K mouse cDNA clone set (http://lgsun.grc.nia.nih.gov/cDNA/15k.html (Tanaka et al. 2000) and spotted in duplicate were purchased from the Human Genome Mapping Project Resource Centre (Hinxton, United Kingdom; http://www.hgmp.mrc.ac.uk/). For each hybridization, equal amounts of fluorescently labeled cDNA were mixed with 4 μg polyA DNA carrier and 6 μg mouse Cot1 DNA (Invitrogen) and subsequently denatured by boiling in hybridization buffer [5× sodium chloride/sodium citrate (SSC); 6× Denhard’s solution; 60 mM Tris, pH 7.6, 0.12% sarcosyl; 48% formamide]. After cooling, 40 μL hybridization mix was applied to the microarray slide, and hybridization was carried out at 50°C for 12–24 hr in a humid hybridization chamber. After hybridization, slides were washed on a shaker at 55°C for 10 min with 2× SSC, 0.2% sodium dodecyl sulfate; 10 min with 2× SSC; 10 min with 0.2× SSC; 1 min with ultrapure water; and 1 min with isopropanol and subsequently dried in a centrifuge for 5 min at 500 × *g*. For embryos, four separate hybridizations [embryo microarray (EM) 1 through 4] addressed both biological and technical variation, using independent samples for EM1, EM2, and EM3/4, and dye reversal of the same samples (based on 12 litters) for EM3 and EM4. For cells, four separate hybridizations [cell microarray (CM) 1 through 4] intentionally addressed only technical variation with duplicate dye reversal. Control samples were labeled with Cy3 for the odd-numbered hybridizations (i.e., EM1, EM3, CM1, CM3) and with Cy5 for the even-numbered ones.

### Microarray Data Analysis

We acquired fluorescent images of microarray slides using a ScanArray confocal laser microarray scanner (Packard BioChip Technologies, Billerica, MA, USA). We quantified fluorescence intensities for the Cy3 and Cy5 channels using the Spot 2.0 software package (Jain et al. 2002). We did not perform background subtraction, as it did not improve the data set. For each spot the base 2 logarithm (log_2_) ratios between the two channels were used to quantify the fold change in relative gene expression levels between experimental and control samples. To remove systematic sources of variation, we used a within-print group scaled normalization method (Yang et al. 2002). A mean value for the duplicate spots was calculated for each array. A parametric empirical Bayes approach (Lönnstedt and Speed 2002) was used to identify differentially expressed genes. The *p*-value was fixed at 0.01, and differentially expressed genes were defined as genes with an absolute log of odds score value above 1. Because no spots were excluded in the analysis (e.g., flagged for morphologic or other defects), and the Bayes approach penalizes for both low absolute expression ratio and high variance between duplicate spots on the same or replicate slides, false negatives may result from a bad spot on one of the four slides. To recover such clones, we repeated the analysis, omitting the four arrays one by one. Genes with an absolute log odds > 1 in any analysis were included in the total list of genes. Hierarchical clustering with complete linkage and Euclidian distance as the distance metric was computed using J-Express 2.1 (Dysvik and Jonassen 2001). In the hierarchical clustering, the outlying value for any gene identified by the leave-one-out procedure (above) was replaced in the omitted array by the median based on the three remaining arrays.

### Quantitative Real-Time RT-PCR

Six genes identified by microarray analysis were selected for reanalysis by quantitative real-time RT-PCR (qPCR). We selected the supposed housekeeping gene peptidylpropyl isomerase A (*Ppia*), also known as cyclophilin, as the endogenous reference because our microarray analysis indicated that its expression is unlikely to be altered by VPA in either mouse embryos or P19 cells. The average log_2_ fold change for *Ppia* (represented by five clones in duplicate in the NIA 1 array) was −0.08 for embryos and −0.04 for cells (data not shown). Moreover, this gene was previously used as endogenous reference for gene expression analysis at the RNA level in the context of VPA and NTDs (Wlodarczyk et al. 1996). Primers were designed with Primer Express software (Applied Biosystems, Palo Alto, CA, USA), using default setting for the TaqMan mode, and synthesized by Applied Biosystem. Primer sequences are given in Table 1. For qPCR, 2 μg total RNA was reverse transcribed in a final volume of 100 μL using TaqMan Reverse Transcription Reagents (Applied Biosystems) with random hexamer primers according to manufacturer instructions. Reactions excluding MultiScribe Reverse Transcriptase (Applied Biosystems) were performed as negative controls. cDNA targets at a 100-fold final dilution were amplified in replicate wells (four for target genes and six for the endogenous reference), using optimized primer concentrations (Table 1) in 1× qPCR Mastermix Plus for SYBR Green I (Eurogentec, Seraing, Belgium) in an ABI Prism 7700 Sequence Detector System (Applied Biosystems) with the following thermal profile: 50°C for 2 min, 95°C for 10 min, followed by 40 cycles of 15 sec at 95°C and 1 min at 60°C. Standard curves for each gene were obtained by amplifying (in quadruplicate) 10-fold serial dilutions of a reference mixture containing 25% each of cDNA derived from VPA-treated and control embryos and cells. Outlying cycle to threshold (C_T_) values were detected using median absolute deviation (Young et al. 2003) with an arbitrary threshold > 10, leading to the removal of one data point. Using the standard curves, C_T_ values for target genes were converted to relative input amounts and normalized to the corresponding values for *Ppia*. Differences in the mean of normalized relative input amounts between VPA-treated and control samples were tested for statistical significance using a two-tailed *t*-test.

## Results

**Figure 1:**
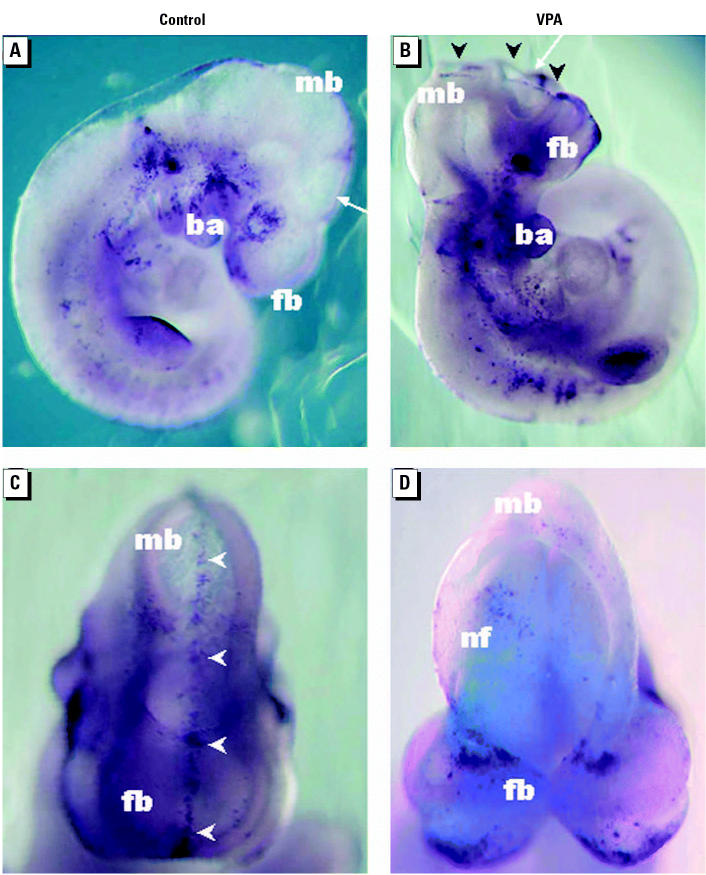
NTDs and apoptosis in VPA-exposed mouse embryos. Whole 10-dpc embryos, stained with the TUNEL technique, viewed from the right (*A,B*) and front (*C,D*). Abbreviations: ba, first branchial arch; fb, forebrain; mb, midbrain; nfs, neural folds. Control (*A,C*) and VPA-treated embryos (*B,D*) were removed (48 hr posttreatment) from the uteri of NMRI dams after ip administration of sodium valproate (600 mg/kg body weight) on 8.0 dpc. Note that VPA-exposed embryos exhibit unfused neural folds, resulting in apparent signs of failed anterior neural tube closure (black arrowheads in *B*). Apoptotic cells (dark) are seen along the line of neural fold fusion in control embryos (white arrowheads in *C*) but not in VPA-exposed embryos (*D*), where instead a transversal band of apoptotic cells can be seen in the neuroepithelium of the forebrain (*D*). Angles of views in *C* and *D* are indicated by white arrows in *A* and *B*, respectively.

To monitor gene expression changes associated with VPA teratogenicity, we adopted conditions of early exposure previously reported to induce NTDs in approximately 60% of live fetuses in the NMRI strain, as observed 18 dpc (Nau and Löscher 1986). By administering a single dose of sodium valproate (600 mg/kg body weight) ip to 8.0-dpc pregnant NMRI dams and examining embryos for developmental defects 48 hr posttreatment (10.0 dpc), we found that 22 of 42 embryos (52%) from VPA-treated dams had different degrees of NTDs, mostly coupled with growth retardation; 15 (36%) were growth retarded but appeared otherwise morphologically normal; and 5 (12%) had diverse abnormalities such as absence of caudal structures, cardiac dysfunction (no heartbeats), and edema. In contrast, we found that no control embryos from saline-treated dams had NTDs or other apparent developmental anomalies. Unlike several other NTD-inducing teratogens (Mirkes 2002), we found that VPA induced no apparent increase in apoptosis along the tips of the neural folds (Copp et al. 2003), as detected by TUNEL staining (Figure 1). Instead, we found a transversal band of apoptotic cells in the forebrain neuroepithelium of VPA-treated embryos (Figure 1D), which to our knowledge has not been reported previously and is the subject for further investigation.

### Valproic Acid–Associated Gene Expression Changes in Mouse Embryos

**Figure 2:**
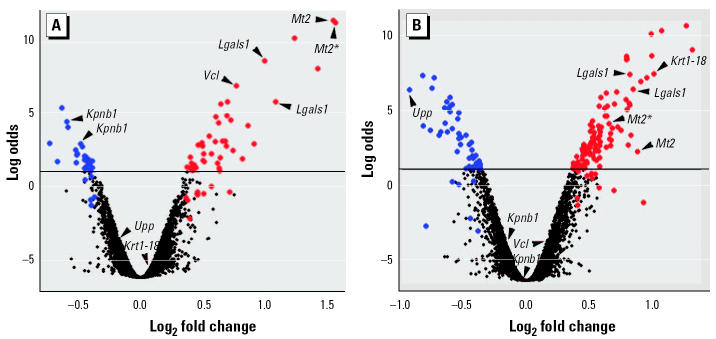
Microarray analysis of transcriptional response to VPA in (*A*) mouse embryos and (*B*) P19 mouse embryocarcinoma cells. The log posterior odds for each clone to be differentially expressed are plotted against the log_2_ fold change of expression for all cDNA clones in the NIA array 1 (see “Materials and Methods”), based on analysis including four replicate microarray slides. The horizontal line marks the threshold (log odds > 1) for selection of a clone as differentially expressed. Upregulated clones are labeled red, and downregulated clones are labeled blue. The clones under the threshold line labeled red or blue were selected (log odds > 1) by leaving out either one of the four replicate slides from the analysis (see “Materials and Methods”). Arrows indicate clones representing the six genes *Kpnb1*, *Krt1-18*, *Lgals1*, *Mt2*, *Upp*, and *Vcl* selected for reanalysis by qPCR (Figure 3). (*A*) Transcriptional response in 8.25-dpc embryos (6 hr posttreatment) from pregnant NMRI mice after ip administration of sodium valproate (600 mg/kg body weight) on 8.0 dpc. (*B*) Transcriptional response in P19 cells cultured in the presence of 1 mM sodium valproate for 24 hr.

To study the gene expression response to VPA during the susceptible stages, that is, when VPA exerts most of its teratogenic effect on the developing neural tube, we extracted total RNA from pools of whole embryos removed from control and VPA-treated 8.25-dpc NMRI mice (6 hr posttreatment) and subjected them to replicated microarray analysis. To identify differentially expressed genes, we used an empirical Bayes model (Lönnstedt and Speed 2002) to rank genes by their log posterior odds of differential expression (Figure 2A). We found that 81 clones of the 6,144 cDNAs from the NIA 15 K mouse cDNA clone set (Tanaka et al. 2000) represented in the NIA array 1 were expected (log odds > 1) to be upregulated (51 of 81) or downregulated (30 of 81) in response to VPA [Supplemental Material, Table 1 (http://ehp.niehs.nih.gov/txg/members/2004/7034/supplemental.pdf)]. An additional 14 clones were selected after the leave-one-out procedure described in “Materials and Methods” [Figure 2A; Supplemental Material, Table 2 (http://ehp.niehs.nih.gov/txg/members/2004/7034/supplemental.pdf)]. Among the wide variety of putative VPA-responsive genes thus listed, we found that metallothionein 2 (*Mt2*) was represented by both top-ranked clones (Supplemental Material, Table 1). Similarly, galectin-1 (*Lgals1*) appeared to be represented by three clones; karyopherin β1 (*Kpnb1*) and H3 histone 3B (*H3f3b*) were represented by two clones each. Approximately one-third of the selected clones appeared to represent uncharacterized or unknown genes (Supplemental Material, Tables 1 and 2). Although the identified candidate genes belong to several functional categories, those encoding matrix/structural proteins appeared to be slightly overrepresented, comprising 35% of the functionally annotated clones (Supplemental Material, Tables 1 and 2).

### Valproic Acid–Associated Gene Expression Changes in P19 Embryocarcinoma Cells

Genes that respond transcriptionally to VPA in embryos (Supplemental Material, Tables 1 and 2) may provide not only important clues about mechanisms of VPA action but also potential biomarkers of VPA teratogenicity that could be exploited in a cell-based screening system. Toward this goal, we employed the pluripotent P19 mouse embryocarcinoma cell line (McBurney 1993) as a possibly relevant cell model for early embryos. To identify general VPA-responsive genes at a dose level close to the range of therapeutic and teratogenic concentrations while attaining convenient and supposedly robust bioassay conditions, total RNA was extracted from P19 cells cultured in the presence or absence of 1 mM VPA for 24 hr and subjected to replicated microarray analysis. Ranking the genes by their log posterior odds of differential expression (Figure 2B), we found 168 clones expected (log odds > 1) to be upregulated (114 of 168) or downregulated (54 of 168) in response to VPA [Supplemental Material, Table 3 (http://ehp.niehs.nih.gov/txg/members/2004/7034/supplemental.pdf)], with 16 additional clones selected by the leave-one-out procedure [Figure 2B; Supplemental Material, Table 4 (http://ehp.niehs.nih.gov/txg/members/2004/7034/supplemental.pdf)]). Again, approximately one-third of the selected clones appeared to represent uncharacterized or unknown genes (Supplemental Material, Tables 3 and 4). Although less apparent than for embryos (Supplemental Material, Tables 1 and 2), genes encoding matrix/structural proteins again represented the largest functional category, comprising 28% of the functionally annotated clones (Supplemental Material, Tables 3 and 4). The similar magnitude of change in cells (Figure 2B; Supplemental Material, Tables 3 and 4) and embryos (Figure 2A; Supplemental Material, Tables 1 and 2) for VPA-responsive genes may support the relevance of the VPA dose (1 mM) used *in vitro*.

### Confirmation of Valproic Acid–Responsive Genes

**Figure 3:**
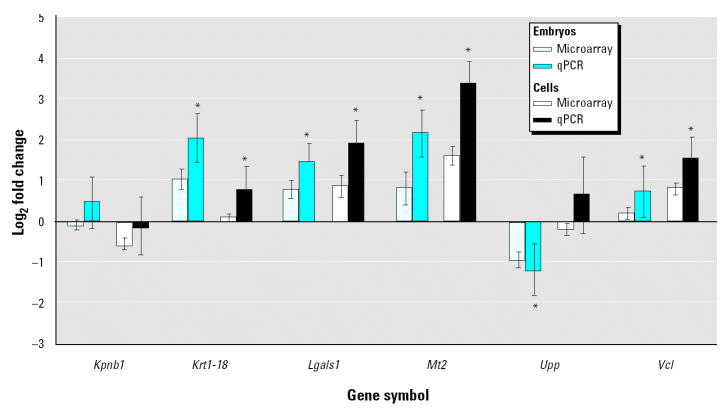
Comparison of log_2_-transformed expression ratios in embryos and P19 cells as determined by microarray analysis and qPCR.
*Significant difference between VPA-treated and control, *p* < 0.05.

To independently assess the altered expression of genes identified by microarray analysis, we arbitrarily selected six genes that appeared biologically relevant while showing diverse responses to VPA in the two-model systems (Figure 2). Using qPCR, we found that the selected genes expected to be upregulated by VPA in embryos [*Lgals1*, *Mt2*, and vinculin (*Vcl*)] or cells [keratin 1-18 (*Krt1-18*), *Lgals1*, and *Mt2*] were significantly (*p* < 0.05) induced (Figure 3). Similarly, the gene expected to be downregulated by VPA in cells [uridine phosphorylase (*Upp*)] was significantly (*p* < 0.05) repressed (Figure 3). Unlike the microarray analysis, using qPCR we could detect a weak but significant (*p* < 0.05) induction of *Krt1-18* and *Vcl* in embryos and cells, respectively (Figure 3). The microarray analysis appeared to underestimate the fold change of expression compared with qPCR (Figure 3), with the only exception being *Kpnb1*, for which a downregulation in embryos was not supported by qPCR (Figure 3).

### Identification of Potential Biomarkers of Valproic Acid Teratogenicity

Genes that respond similarly to a teratogen in a cultured cell model as in intact embryos might be directly exploited as biomarkers in an *in vitro* test system, using the same cell line. To identify candidates for such potential biomarkers, we compared the results presented in Supplemental Material, Tables 1–4, and found 29 clones (three of which were recovered in embryos by the leave-one-out procedure) likely to be VPA responsive in both embryos and P19 cells (Table 2). These clones probably represent no more than 25 genes, of which 16 currently have known identity (Table 2). Among these genes are several that were top ranked in embryos (Supplemental Material, Table 1), such as *Mt2*, metallothionein 1 (*Mt1*), *Lgals1*, *H3f3b*, creatine kinase–brain (*Ckb*), and transgelin 2 (*Tagln2*). The similar transcriptional response to VPA in the cell model as in embryos strengthens the case not only for these genes as VPA targets in the embryo but also for a number of other genes (Table 2; Supplemental Material, Tables 1 and 2) such as cytochrome c oxidase subunit VIIa polypeptide 2-like (*Cox7a2l*), ubiquitin carboxy-terminal hydrolase L1 (*Uchl1*), eukaryotic translation initiation factor 4 gamma 2 (*Eif4g2*), bromodomain containing protein 4 (*Brd4*), annexin A11 (*Anxa11*), leukotriene B4 12-hydroxydehydrogenase (*Ltb4dh*), inhibitor of DNA binding 1 (*Idb1*), and fatty acid synthase (*Fasn*).

**Figure 4:**
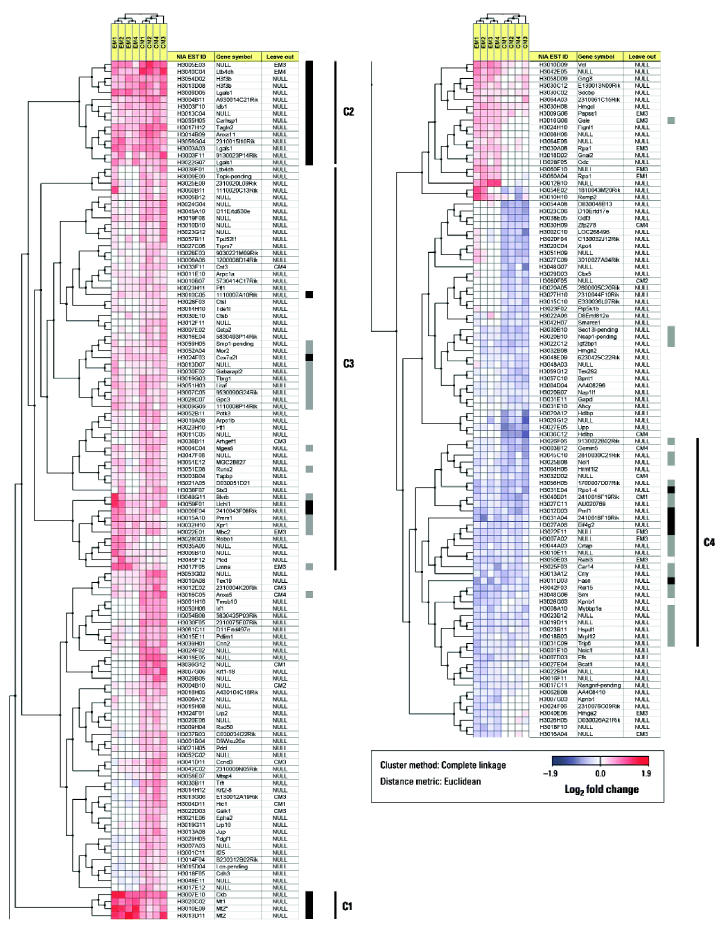
Hierarchical two-way clustering of all 220 genes expected to be transcriptionally responsive to VPA (log odds > 1) in either embryos (Supplemental Material, Tables 1 and 2) or cells (Supplemental Material, Tables 3 and 4), using the mean log_2_ fold change of expression (represented by a blue–red color scale; bottom) on both sets of four replicate microarray slides (embryos: EM1–EM4 and cells: CM1–CM4; see “Materials and Methods”). The thick vertical lines to the right of the heat map mark the 29 clones with log odds > 1 that respond similarly to VPA in both embryos and cells (black), and the additional 41 clones with log_2_ fold changes > 0.3 or < −0.3 in both embryos and cells (gray). At the right, four discernible clusters (C1–C4) are marked with thin vertical lines.

As we used a cutoff for differential expression intended to minimize the number of false positives, the number of genes with a similar transcriptional response to VPA in the cell model as in embryos might be underestimated in our analysis. By clustering the mean log_2_ fold changes measured in the eight individual microarray slides for all 220 selected clones (i.e., clones with log odds > 1 in either embryos or cells), we found that most of the clones displayed in Table 2 form two well-defined clusters of commonly upregulated genes (clusters C1 and C2), whereas the rest of the clones are found within two indistinct clusters of commonly upregulated (cluster C3) or downregulated (cluster C4) genes (Figure 4). Across all 6,144 clones, we found that the highest log odds score for which the transcriptional change in embryos was not in the same direction as in cells (disregarding the magnitude of change) was −0.57. Among the 122 clones above this level in embryos, the highest and lowest log_2_ fold changes detected were 0.33 and −0.31, respectively. Applying log_2_ fold change > 0.3 and < −0.3 (corresponding to > 27% fold change) as the cutoff, we could identify 41 additional clones as putative candidates for genes with a similar transcriptional response to VPA in the cell model as in embryos (Figure 4).

### Rapid Valproic Acid–Induced Transcriptional Activation of the Genes Encoding Galectin-1 and Vinculin

**Figure 5:**
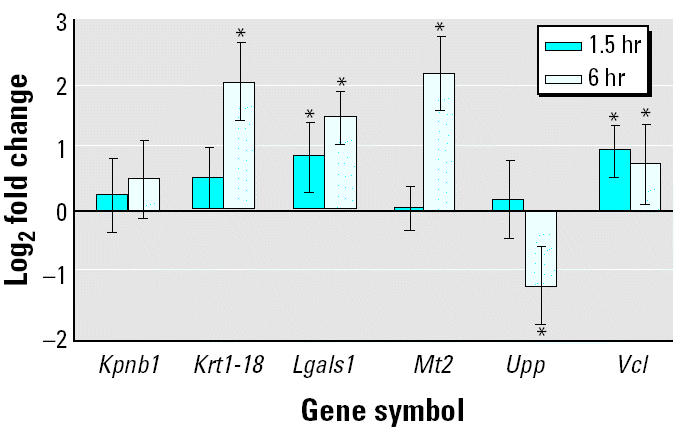
Transcriptional response in embryos 1.5 and 6 hr posttreatment after ip administration of sodium valproate (600 mg/kg body weight) on 8.0 dpc, as determined by qPCR.
*Significant difference between VPA-treated and control, *p* < 0.05.

Toward understanding the mechanism of VPA action and validating candidate genes as potentially useful biomarkers of VPA responses, we reinvestigated the VPA-induced transcriptional response of selected genes (Figure 2) in mouse embryos. VPA reaches peak levels in mouse serum about 30 min after a single ip injection, and the half-time of VPA clearance from mouse serum is about 1 hr (Nau et al. 1991). To investigate whether VPA at such a peak concentration can induce a rapid transcriptional response of selected genes, we extracted total RNA from pools of whole embryos removed from control and VPA-treated pregnant NMRI dams 1.5 hr posttreatment (dose and gestational day of treatment as before) and subjected to qPCR. Among the six tested genes, the expression of *Lgals1* and *Vcl* were significantly (*p* < 0.05) induced after 1.5 hr (Figure 5), which may support independently the VPA responsiveness of these two genes.

## Discussion

Although several modes of VPA action have been proposed [e.g., histone deacetylase (HDAC) and protein kinase (PKC) inhibition, extracellular signal-regulated kinase (ERK) and activator protein-1 (AP-1) activation, and effects on the actin cytoskeleton] (Blaheta and Cinatl 2002; Gurvich and Klein 2002; and Walmod et al. 1999), the mechanism of VPA teratogenicity remains poorly understood. In this study we used microarrays to monitor gene expression changes in response to VPA during stages critical to VPA-induced NTDs in the mouse embryo. Some of the more than 70 putative VPA target genes thus identified (Supplemental Material, Table 1) have previously been directly or indirectly linked to VPA effects or to NTDs or processes relevant to neural tube formation and closure, but most appear to be novel candidates. Moreover, we propose that many of these genes by virtue of their similar expression changes in embryos and cultured P19 mouse embryocarcinoma cells (Table 2; Figure 4) could be directly exploited as potential biomarkers of VPA action in cell-based assays.

To date, mouse embryos have not been extensively studied by microarray analysis (Carter et al. 2003; Smith and Greenfield 2003), partly because of their small size and limited RNA content. Here we overcome this limitation by pooling whole embryos from several similarly treated mice. Despite our primary goal to identify candidate genes that may be VPA targets in disturbed neural tube closure, the use of whole embryos rather than isolated neural tubes may be warranted for at least three reasons. First, VPA accumulates in the neuroepithelium (Dencker et al. 1990), which constitutes a major region of the mouse embryo during the stages investigated. Second, neural tube closure can be influenced by genes that are mostly or only expressed outside the neural tube [e.g., cartilage homeoprotein 1 (*Cart1*), *Twist,* and sonic hedgehog (*Shh*)] (Copp et al. 1990). Third, potential dissection artifacts (Diaz et al. 2003) are minimized. In addition, bulk approaches such as the pooling strategy we used in this study may be warranted to allow the study of gene expression changes during early stages of neural tube development without the need to know which individual embryos will subsequently develop NTDs in response to the VPA treatment (50–60%). An unfavorable consequence of pooling whole embryos from multiple litters, however, is that we dilute the expression changes for those genes expressed only in certain defined regions of the embryo, such as distinct areas of the neural tube, as well as for any genes that may be responding to VPA mostly or only in those embryos that will become malformed. To some degree, this may account for why we are able to identify fewer differentially expressed genes in the embryo (Supplemental Material, Tables 1 and 2) than in the P19 cell line (Supplemental Material, Tables 3 and 4).

The formation and closure of the neural tube is a highly coordinated set of events that involves a multitude of morphogenetic movements and regulated cell behavior (Copp et al. 2003; DeSesso et al. 1999; Smith and Schoenwolf 1997). Several null mutations for actin-regulating genes have been reported to be associated with NTDs (Copp et al. 2003), illustrating that proper regulation of cell shape and cell movements is crucial for neurulation processes to occur normally. In this study, we found that the expression of the gene encoding *Vcl* is rapidly induced in embryos after VPA treatment (Figure 5). Vinculin, which is essential for neural tube closure (Xu et al. 1998), is an actin-binding protein associated with focal adhesions (De Arcangelis and Georges-Labouesse 2000) and has previously been reported to be increased in such points of integrin-mediated cell–matrix interactions after VPA treatment *in vitro* (Walmod et al. 1999). Some integrins and extracellular matrix components, along with components downstream of integrin signaling, also appear to be essential for neural tube closure (Brouns et al. 2000; De Arcangelis and Georges-Labouesse 2000; Juriloff and Harris 2000). Adverse effects of VPA on the dynamics of the actin cytoskeleton may therefore contribute to VPA teratogenicity, as has been previously suggested (Walmod et al. 1999). The supposed actin dependency of anterior but not posterior neuropore closure in mouse embryos (Ybot-Gonzalez and Copp 1999) could thus explain why exencephaly is the dominant NTD observed in VPA-exposed mice (Nau et al. 1991). Although emerging as a conceivable VPA target from a mechanistic point of view, vinculin might be less straightforward to exploit as a biomarker of VPA effects given the weak response in our cell model (Figure 3). Conversely, we found that the expression of the gene encoding *Tagln2*, an actin-binding protein with the ability to cross-link actin filaments (Shapland et al. 1993), was induced, and the genes encoding spermidine synthase (*Srm*), an actin-regulating protein (Caruso et al. 1994), and thyroid hormone receptor interactor 6 (*Trip6* ), a focal adhesion-binding protein with nuclear shuttling activity, were repressed in response to VPA in both embryos and P19 cells (Table 2; Figure 4). Our findings may support a role of integrin-mediated actin regulation in VPA teratogenicity, even though we were unable to observe any apparent reorganization of the actin cytoskeleton, as visualized by phalloidin staining (data not shown), in P19 cells treated with VPA at the present concentration (1 mM).

Along these lines, we found the expression of the gene *Lgals1*, which encodes the β-galactoside–binding protein galectin-1 (Barondes et al. 1994), was induced by VPA in both embryos and cells (Table 2; Figure 4). Galectin-1 is a multifunctional homodimeric lectin whose extracellular and intracellular activities are thought to regulate cellular processes as diverse as cell–matrix interactions, signal transduction, migration, differentiation, proliferation, apoptosis, and RNA splicing (Hughes 2001; Liu et al. 2002; Perillo et al. 1998). Binding of galectin 1 to the extracellular portion of β1 integrin (Moiseeva et al. 2003) may modulate cell adhesion to extracellular matrix components such as fibronectin and laminin and activate downstream events of integrin signaling (Giancotti and Ruoslahti 1999). Intracellularly, galectin-1 appears to be recruited by the G-protein H-Ras, a membrane-associated transducer of integrin and receptor tyrosine kinase signaling (Giancotti and Ruoslahti 1999), to stabilize its active guanosine triphosphate (GTP)-bound state and membrane anchorage in microdomains segregated from lipid rafts (Hancock 2003). Overexpression of galectin-1 and its binding to H-Ras-GTP may thus enhance signaling through Raf1 and the ERK pathway (Hancock 2003), which along with the downstream effector AP-1 is activated by VPA (Blaheta and Cinatl 2002; Yuan et al. 2001).

The rapid activation of the *Lgals1* gene (Figure 5) indicates that VPA may act at the immediate level of the *Lgals1* promoter. Because VPA is a direct HDAC inhibitor (Göttlicher et al. 2001; Phiel et al. 2001) and because other HDAC inhibitors such as butyrate and trichostatin A (TSA) induce galectin-1 expression at the transcriptional level (Lu and Lotan 1999), it is likely that VPA induces expression at the *Lgals1* promoter by virtue of its activity as an HDAC inhibitor. As a short-chain carboxylic acid structurally related to VPA, butyric acid has been reported to be teratogenic in whole-embryo culture (Coakley et al. 1986), and the structurally unrelated TSA has been reported to induce NTDs in mouse embryos *in vitro* (Svensson et al. 1998). *Hdac1*^−/−^mouse embryos die severely growth retarded before 10.5 dpc, and *Hdac1*^−/−^ embryonic stem cells proliferate poorly, indicating that the silencing of gene expression by Hdac1 is essential for cell proliferation during embryonic development (Lagger et al. 2002). As shown for other HDAC inhibitors, the effects on gene expression by VPA may depend on the transcription factor specificity protein 1 (Sp1) (Arinze and Kawai 2003), which binds to GC-rich promoter elements (Suske 1999). The DNA-binding activity of Sp1 is modulated by direct interaction with HDAC (Doetzlhofer et al. 1999), and it has been suggested that VPA and TSA inhibit the activity of HDAC by interfering with its catalytic site (Göttlicher et al. 2001). Butyrate may affect Sp1-mediated induction of *Lgals1* transcription by a mechanism, as yet unknown, distinct from that of TSA (Lu and Lotan 1999). It is therefore tempting to speculate that VPA, by virtue of its known blocking of TSA binding to HDAC (Göttlicher et al. 2001) and structural similarity to butyric acid, could act by either or both of these mechanisms to induce gene expression at the *Lgals1* promoter. Hence, the activation of the ERK/AP-1 pathway by VPA could occur downstream of HDAC inhibition and Sp1-induced galectin-1 overexpression.

The transcriptional induction we found in embryos and P19 cells (Table 2; Figure 4) of the genes encoding the metal-regulating proteins *Mt1* and *Mt2* may also be attributed to the HDAC-inhibitory activity of VPA (Marks et al. 2003), as these genes may be activated through Sp1 derepression (Ogra et al. 2001) or chromatin-opening histone acetylation (Ghoshal et al. 2002). Butyrate increases mRNA levels of both these genes in embryocarcinoma cell lines (Andrews and Adamson 1987), and VPA increases the level of metallothionein protein in the liver of mice (Kaji and Mikawa 1991) and pregnant rats (Bui et al. 1998; Keen et al. 1989), causing zinc depletion in the embryos. In the present study we found induced metallothionein expression also in the embryo after maternal VPA administration (Table 2), possibly exacerbating the depletion of Zn^2+^ available for developmental processes. It is conceivable that disturbed Zn^2+^ availability could affect, among other processes, the activity of zinc finger–containing transcription factors essential for normal neural tube development, such as Yin Yang 1 (YY1) (Donohoe et al. 1999) and Zic1 through Zic3 (Klootwijk et al. 2000; Nagai et al. 1997, 2000). Because of this evidence and the association between Zn deficiency and human NTDs (Zimmerman 1984), disrupted Zn homeostasis may appear as an attractive mechanism for VPA teratogenesis despite some evidence against Zn deficiency as the cause of VPA-induced exencephaly in the mouse embryo (Wegner et al. 1990). Copper depletion could also be the culprit because metallothionein binds Cu^2+^ more strongly than Zn^2+^ (Holt et al. 1980), VPA treatment enhances copper excretion (Kuzuya et al. 2002), and knockout of the copper transporter *Ctr1* results in NTDs (Lee et al. 2001). An obvious weakness with the concept of VPA teratogenesis being mediated by metallothionein induction is that *Mt1* and *Mt2* are coordinately induced by such a wide variety of stressors (Andrews 2000) that they could reasonably be dismissed as being part of a general stress response (Brady 1981) rather than being linked to a specific compound such as VPA. However, exposure to an HDAC inhibitor may not be automatically stressful in this regard. TSA alone, for example, activates the *MT1* promoter in some cell types (Dressel et al. 2000) but not in others (Ghoshal et al. 2002).

Metallothioneins are also potent antioxidants (Andrews 2000). The role of reactive oxygen species (ROS) in developmental toxicity is well documented (Fantel 1996). Their role in VPA teratogenicity, however, remains unclear, although ROS production has been detected in response to VPA *in vitro* (Na et al. 2003), and VPA-induced NTDs are prevented by the antioxidant vitamin E *in vivo* (Al Deeb et al. 2000). It is therefore interesting that we found the gene *Ltb4dh*, which encodes a protein with antioxidant properties (Dick et al. 2001), induced by VPA in both embryos and P19 cells (Table 2).

In addition to the known developmental importance of a gene, as determined by the phenotypes of knockout mice, a gene’s putative involvement in neural tube development may also be inferred from its expression domain. For example, we find a reduced expression of the gene *Fasn* in both embryos and P19 cells (Table 2). This gene is normally expressed in developmentally active regions such as the dorsal neural folds of the closing neural tube (Chirala et al. 2003). If Fasn protein is crucial for these processes, VPA-induced downregulation of *Fasn* gene expression could evidently disturb them. Considering that homozygous *Fasn* knockout mice die before implantation and also that most of the *Fasn*^+/−^ mice die or develop abnormally (Chirala et al. 2003), *Fasn* emerges as a putative target for teratogen action. Intriguingly, the *Fasn* gene is transcriptionally regulated by Sp1 (Fukuda et al. 1999), again pointing toward HDAC inhibition as a potential cause for the VPA responsiveness.

Another way by which a gene may be associated with NTDs is if its product acts in a pathway essential for neural tube development. Bone morphogenetic protein (BMP) signaling is likely to represent such a pathway, given that knockout of both the BMP signaling transducer Smad5 (Chang et al. 1999) and the BMP inhibitor noggin (McMahon et al. 1998) results in NTDs (mice homozygous for null alleles of the genes encoding BMP-2, BMP-4, and BMP receptor type IA die before or around neurulation). Members of the inhibitor of DNA binding family, which are dominant negative regulators of basic helix–loop–helix transcription factors with diverse cellular effects, are among the most important downstream targets and effectors of BMP signaling (Miyazono and Miyazawa 2002). The enhanced expression of the *Idb1* gene we observed in both VPA-treated embryos and cells (Table 2) could therefore reflect or mimic disturbed BMP signaling. *Idb1* gene activation by Smads is inhibited by YY1 (Kurisaki et al. 2003), the repressive activity of which may be mediated by interactions with HDAC1 (Yang et al. 1996) and Sp1 (Lee et al. 1993) through a GC-rich Sp1/YY1-binding enhancer site in the *Idb1* gene (Lopez-Rovira et al. 2002). HDAC inhibition, by relieving both Sp1 and YY1 repression, may therefore cause dysregulation of *Idb1* gene activity.

It is striking that so many of the identified VPA-responsive genes encode for proteins that are multifunctional or have integrative activities, galectin-1 being an obvious example. Similar to galectin-1, members of the annexin family have intracellular, extracellular, and membrane-bound functions and are involved in cell–matrix interactions, cell growth, and differentiation (Seaton and Dedman 1998). The VPA-induced transcriptional induction of annexin A5 (*Anxa5*) and *Anxa11* we detect in both embryos and cells (Table 2) may depend on HDAC inhibition, as the mouse *Anxa5* and the human *ANXA11* genes have Sp1 sites in their promoters (Bances et al. 2000; Rodriguez-Garcia et al. 1999). Interestingly, annexins may be substrates for and negatively regulate phosphatidylinostiol-dependent PKC activity, the inhibition of which is an established VPA effect (Blaheta and Cinatl 2002). Kpnb1 (also known as importin-β) may also be categorized as multifunctional, as it is a nuclear transport factor with additional roles as a chaperone in the cytoplasm and during mitosis (Jäkel et al. 2002). Although *Kpnb1* is a gene whose expression has been reported to be downregulated by HDAC inhibition (Marks et al. 2003), our present data do not provide conclusive evidence for its repression by VPA in mouse embryos (Supplemental Material, Table 1; Figure 3). Trip6 is another good example of a protein with dual localization and function. In addition to its association with focal adhesions, it functions as an intracellular signaling molecule that shuttles between the cell surface and the nucleus (Wang et al. 1999), where it acts as a transcription factor.

Recently, the expression of *Trip6*, along with *Eif4g2*, and particularly *Upp*, was reported to be stem cell specific (Ramalho-Santos et al. 2002). Essentially, these genes were identified as part of a set of genes defining “stemness,” that is, promotion of cell self-renewal and suppression of differentiation. It is therefore interesting that we found the expression of *Eif4g2* and *Trip 6* downregulated in response to VPA in P19 cells and embryos (Table 2; Figure 4) and the expression of *Upp* downregulated in P19 cells (Figure 3; Supplemental Material, Table 4). Similarly, Krt1-18 has been reported as a marker of stem cell differentiation (Kelly and Rizzino 2000). Increased expression of the *Krt1-18* gene has recently been reported to be a marker for VPA-induced differentiation in F9 mouse embryocarcinoma cells (Werling et al. 2001), a cell line similar to P19 cells. An Sp1 site in the *Krt1-18* promoter is important for the expression of this gene (Gunther et al. 1995), which is also activated by butyrate and TSA in F9 cells (Miyashita et al. 1994), suggesting that VPA may activate *Krt1-18* expression through HDAC inhibition. Our data support that *Krt1-18* and the co-regulated *Krt2-8* gene may be VPA inducible in cultured embryocarcinoma cells (Figure 3; Supplemental Material, Table 2) and that *Krt1-18* may respond weakly in the embryo (Figure 3).

We conclude that microarray-based toxicogenomics approaches may be useful for identifying target genes and biomarkers of developmental toxicity. By linking gene expression changes to toxic outcome, we detected alterations in gene expression at the level of whole embryos that may be further investigated in terms of hypotheses about mechanisms underlying defective neural tube development (*Vcl*, *Tagln2*, *Trip6*, *Mt1*, *Mt2*, *Fasn*, *Id1*). By comparing gene expression changes in whole embryos with those in a cultured cell model, we defined a subset of VPA-responsive genes that may be evaluated as potential biomarkers of VPA teratogenicity (*Lgals1*, *Id1*, *Fasn*, *Anxa5*). A recurrent theme among these genes, as well as for others (*Mt1*, *Anxa11*, *Krt1-18*), is that they may be activated or repressed through HDAC inhibition and Sp1 activation, indicating that HDAC may be a primary molecular target of VPA action *in vivo*. It remains to be determined to what extent the disruptive effect of VPA on neural tube development may be compounded from the deregulation of a wide variety of target genes acting downstream of HDAC inhibition. Our toxicogenomics approach provides a framework for further studies of developmental toxicity induced by VPA and other chemicals, addressing parameters such as dose, time, and duration of exposure, and genetic susceptibility. In summary, the parallel use of *in vivo* and *in vitro* models in conjunction with global expression profiling emerges as a relevant approach toward the identification of biomarkers associated with toxicity after exposure to a wide variety of environmental teratogens.

## Supplementary Material

Supplemental Tables

## Figures and Tables

**Table 1:** PCR primers.

Gene symbol	Forward primer sequence	Forward primer concentration (nM)	Reverse primer sequence	Reverse primer concentration (nM)	Amplicon length (nt)
*Kpnb1*	5′-GGGAATCGTCCAGGGATTG- 3′	900	5′-AAATAAATTCTACTCTGGCTGTACCA- 3′	900	83
*Krt1-18*	5′-AATCGAGGCACTCAAGGAAGAA- 3′	300	5′-GGCATCCACTTCCACGTCA- 3′	300	112
*Lgals1*	5′-GAATCTCTTCGCTTCAGCTTCA- 3′	50	5′-CAGGTTTGAGATTCAGGTTGCT- 3′	50	68
*Mt2*	5′-CGCCATGGACCCAACT- 3′	50	5′-AGGAAGTACATTTGCATTGTTTGC-3′	50	89
*Upp*	5′-TCACCATCATCCGCATTGG- 3′	300	5′-GCCTGCTGCGTGATGACA- 3′	900	73
*Vcl*	5′-TGCCAAGCAGTGCACAGATAA- 3′	50	5′-GGTCCGGCCCAGCATAGT- 3′	50	124
*Ppia*	5′-TTCCTCCTTTCACAGAATTATTCCA- 3′	50	5′-CCGCCAGTGCCATTATGG -3′	50	75

**Table 2:** Genes responding transcriptionally to VPA in embryos and P19 cells.

			Embryos		Cells		
NIA EST ID	Gene symbol*^a^*	Gene name*^a^*	Log odds	Log_2_ fold change	Log odds	Log_2_ fold change	Function*^b^*
H3014B09	*Anxa11*	Annexin A11	2.2	0.58	7.1	0.97	Matrix/structural proteins
H3056G04	*Brd4*	Bromodomain containing 4	2.5	0.76	8.6	0.81	Signal transduction
H3055H05	*Carhsp1*	Calcium-regulated heat stable protein 1	1.1	0.42	3.6	0.59	?
H3007E10	*Ckb*	Creatine kinase, brain	8.2	1.45	10.0	1.01	Matrix/structural proteins
H3024F03	*Cox7a2l*	Cytochrome c oxidase subunit VIIa polypeptide 2-like	3.0	0.50	4.0	0.52	?
H3027A06	*Eif4g2*	Eukaryotic translation initiation factor 4 gamma 2	2.7	−0.48	2.3	−0.44	Protein synthesis/translational control
H3011D03	*Fasn*	Fatty acid synthase	1.7	−0.68	1.7	−0.46	Matrix/structural proteins
H3054D02	*H3f3b*	H3 histone, family 3B	5.9	0.71	8.4	0.81	Transcription/chromatin
H3013D08	*H3f3b*	H3 histone, family 3B	5.7	0.66	6.9	0.92	Transcription/chromatin
H3003F10	*Idb1*	Inhibitor of DNA binding 1	1.8	0.51	5.2	0.65	Transcription/chromatin
H3009D05	*Lgals1*	Lectin, galactose binding, soluble 1	8.7	1.02	6.4	0.86	Matrix/structural proteins
H3003A03	*Lgals1*	Lectin, galactose binding, soluble 1	4.2	0.88	7.4	0.84	?
H3022G07	*Lgals1*	Lectin, galactose binding, soluble 1	3.1	0.67	3.1	0.58	Matrix/structural proteins
H3040C04	*Ltb4dh*	Leukotriene B4 12-hydroxydehydrogenase	1.8	0.69	9.0	1.34	?
H3020C02	*Mt1*	Metallothionein 1	10.3	1.26	5.4	0.71	Heat shock/stress
H3013D11	*Mt2*	Metallothionein 2	11.6	1.58	2.2	0.90	Heat shock/stress
H3010E09	Mt2*	Metallothionein 2*	11.4	1.60	4.0	0.67	Heat shock/stress**
H3012D03	*Pmf1*	Polyamine-modulated factor 1	1.6	−0.53	4.1	−0.66	?
H3031E04	*Rpo1-4*	RNA polymerase 1–4	1.8	−0.46	3.5	−0.64	Protein synthesis/translational control
H3017H12	*Tagln2*	Transgelin 2	4.4	0.64	10.3	1.09	?
H3059F01	*Uchl1*	Ubiquitin carboxy-terminal hydrolase L1	2.9	0.93	1.2	0.59	?
H3010C05	1110007A10Rik	RIKEN cDNA 1110007A10 gene	1.3	0.38	1.5	0.41	?
H3031A04	2410016F19Rik	RIKEN cDNA 2410016F19 gene	3.0	−0.74	2.7	−0.46	?
H3009E04	2410043F08Rik	RIKEN cDNA 2410043F08 gene	1.2	0.64	2.6	0.56	?
H3003F11	9130023P14Rik	RIKEN cDNA 9130023P14 gene	1.6	0.63	3.3	0.85	Matrix/structural proteins
H3004B11	A930014C21Rik	RIKEN cDNA A930014C21 gene	2.8	0.51	5.0	0.81	?
H3022F11	?	?	1.9	−0.49	1.2	−0.46	?
H3005E03	?	?	1.6	0.73	10.6	1.29	?
H3013C04	?	?	1.3	0.44	5.1	0.63	?**

Abbreviations: ?, unknown genes and/or functions; EST, expressed sequence tag; ID, identifier.

a Gene symbols are from the NIA web site (http://lgsun.grc.nia.nih.gov/cDNA/15k.html) as of 30 May 2003, except where indicated by asterisk.

b Gene names are from SOURCE (http://source.stanford.edu/cgi-bin/source/SourceSearch) as of 19 September 2003, except where indicated by asterisk.

* Annotation by tBLASTx (http://www.ncbi.nlm.nih.gov/BLAST/).

** Annotation performed manually, i.e., computer-assigned functions of unknown genes were removed, or known functions for genes identified by tBLASTx were added.

## References

[b1-ehp0112-001225] Aardema MJ, MacGregor JT (2002). Toxicology and genetic toxicology in the new era of “toxicogenomics”: impact of “-omics” technologies. Mutat Res.

[b2-ehp0112-001225] Al Deeb S, Al Moutaery K, Arshaduddin M, Tariq M (2000). Vitamin E decreases valproic acid induced neural tube defects in mice. Neurosci Lett.

[b3-ehp0112-001225] Andrews GK (2000). Regulation of metallothionein gene expression by oxidative stress and metal ions. Biochem Pharmacol.

[b4-ehp0112-001225] Andrews GK, Adamson ED (1987). Butyrate selectively activates the metallothionein gene in teratocarcinoma cells and induces hypersensitivity to metal induction. Nucleic Acids Res.

[b5-ehp0112-001225] Arinze IJ, Kawai Y (2003). Sp family of transcription factors is involved in valproic acid-induced expression of galphai2. J Biol Chem.

[b6-ehp0112-001225] Bances P, Fernandez MR, Rodriguez-Garcia MI, Morgan RO, Fernandez MP (2000). Annexin A11 (ANXA11) gene structure as the progenitor of paralogous annexins and source of orthologous cDNA isoforms. Genomics.

[b7-ehp0112-001225] Barondes SH, Cooper DN, Gitt MA, Leffler H (1994). Galectins. Structure and function of a large family of animal lectins. J Biol Chem.

[b8-ehp0112-001225] Blaheta RA, Cinatl J (2002). Anti-tumor mechanisms of valproate: a novel role for an old drug. Med Res Rev.

[b9-ehp0112-001225] Brady FO (1981). Synthesis of rat hepatic zinc thionein in response to the stress of sham operation. Life Sci.

[b10-ehp0112-001225] Brouns MR, Matheson SF, Hu KQ, Delalle I, Caviness VS, Silver J (2000). The adhesion signaling molecule p190 RhoGAP is required for morphogenetic processes in neural development. Development.

[b11-ehp0112-001225] Bui LM, Taubeneck MW, Commisso JF, Uriu-Hare JY, Faber WD, Keen CL (1998). Altered zinc metabolism contributes to the developmental toxicity of 2-ethylhexanoic acid, 2-ethylhexanol and valproic acid. Toxicology.

[b12-ehp0112-001225] Carter MG, Hamatani T, Sharov AA, Carmack CE, Qian Y, Aiba K (2003). *In situ*-synthesized novel microarray optimized for mouse stem cell and early developmental expression profiling. Genome Res.

[b13-ehp0112-001225] Caruso A, Pellati A, Bosi G, Arena N, Stabellini G (1994). Effects of spermidine synthase inhibition on cytoskeletal organization in cultured chick embryo fibroblasts. Eur J Histochem.

[b14-ehp0112-001225] Chang H, Huylebroeck D, Verschueren K, Guo Q, Matzuk MM, Zwijsen A (1999). Smad5 knockout mice die at mid-gestation due to multiple embryonic and extraembryonic defects. Development.

[b15-ehp0112-001225] Chirala SS, Chang H, Matzuk M, Abu-Elheiga L, Mao J, Mahon K (2003). Fatty acid synthesis is essential in embryonic development: fatty acid synthase null mutants and most of the heterozygotes die *in utero*. Proc Natl Acad Sci USA.

[b16-ehp0112-001225] Coakley ME, Rawlings SJ, Brown NA (1986). Short-chain carboxylic acids, a new class of teratogens: studies of potential biochemical mechanisms. Environ Health Perspect.

[b17-ehp0112-001225] Copp A, Brook FA, Estibeiro JP, Shum AS, Cockroft DL (1990). The embryonic development of mammalian neural tube defects. Progr Neurobiol.

[b18-ehp0112-001225] Copp AJ, Greene ND, Murdoch JN (2003). The genetic basis of mammalian neurulation. Nat Rev Genet.

[b19-ehp0112-001225] Craig JC, Bennett GD, Miranda RC, Mackler SA, Finnell RH (2000). Ribonucleotide reductase subunit R1: a gene conferring sensitivity to valproic acid-induced neural tube defects in mice. Teratology.

[b20-ehp0112-001225] De Arcangelis A, Georges-Labouesse E (2000). Integrin and ECM functions: roles in vertebrate development. Trends Genet.

[b21-ehp0112-001225] Dencker L, Nau H, D’Argy R (1990). Marked accumulation of valproic acid in embryonic neuroepithelium of the mouse during early organogenesis. Teratology.

[b22-ehp0112-001225] DeSesso JM, Scialli AR, Holson JF (1999). Apparent lability of neural tube closure in laboratory animals and humans. Am J Med Genet.

[b23-ehp0112-001225] Diaz E, Yang YH, Ferreira T, Loh KC, Okazaki Y, Hayashizaki Y (2003). Analysis of gene expression in the developing mouse retina. Proc Natl Acad Sci USA.

[b24-ehp0112-001225] Dick RA, Kwak MK, Sutter TR, Kensler TW (2001). Antioxidative function and substrate specificity of NAD(P)H-dependent alkenal/one oxidoreductase. A new role for leukotriene B4 12-hydroxydehydrogenase/15-oxoprostaglandin 13-reductase. J Biol Chem.

[b25-ehp0112-001225] Docterman KE, Smith SM (2002). Of meis and men: lessons from a microarray study of teratogen action. Teratology.

[b26-ehp0112-001225] Doetzlhofer A, Rotheneder H, Lagger G, Koranda M, Kurtev V, Brosch G (1999). Histone deacetylase 1 can repress transcription by binding to Sp1. Mol Cell Biol.

[b27-ehp0112-001225] Donohoe ME, Zhang X, McGinnis L, Biggers J, Li E, Shi Y (1999). Targeted disruption of mouse Yin Yang 1 transcription factor results in peri-implantation lethality. Mol Cell Biol.

[b28-ehp0112-001225] Downs KM, Davies T (1993). Staging of gastrulating mouse embryos by morphological landmarks in the dissecting microscope. Development.

[b29-ehp0112-001225] Dressel U, Renkawitz R, Baniahmad A (2000). Promoter specific sensitivity to inhibition of histone deacetylases: implications for hormonal gene control, cellular differentiation and cancer. Anticancer Res.

[b30-ehp0112-001225] Dysvik B, Jonassen I (2001). J-Express: exploring gene expression data using Java. Bioinformatics.

[b31-ehp0112-001225] Faiella A, Wernig M, Consalez GG, Hostick U, Hofmann C, Hustert E (2000). A mouse model for valproate teratogenicity: parental effects, homeotic transformations, and altered HOX expression. Hum Mol Genet.

[b32-ehp0112-001225] Fantel AG (1996). Reactive oxygen species in developmental toxicity: review and hypothesis. Teratology.

[b33-ehp0112-001225] Fukuda H, Noguchi T, Iritani N (1999). Transcriptional regulation of fatty acid synthase gene and ATP citrate-lyase gene by Sp1 and Sp3 in rat hepatocytes(1). FEBS Lett.

[b34-ehp0112-001225] Ghoshal K, Datta J, Majumder S, Bai S, Dong X, Parthun M (2002). Inhibitors of histone deacetylase and DNA methyltransferase synergistically activate the methylated metallothionein I promoter by activating the transcription factor MTF-1 and forming an open chromatin structure. Mol Cell Biol.

[b35-ehp0112-001225] Giancotti FG, Ruoslahti E (1999). Integrin signaling. Science.

[b36-ehp0112-001225] Göttlicher M, Minucci S, Zhu P, Kramer OH, Schimpf A, Giavara S (2001). Valproic acid defines a novel class of HDAC inhibitors inducing differentiation of transformed cells. EMBO J.

[b37-ehp0112-001225] Gunther M, Frebourg T, Laithier M, Fossar N, Bouziane-Ouartini M, Lavialle C (1995). An Sp1 binding site and the minimal promoter contribute to overexpression of the cytokeratin 18 gene in tumorigenic clones relative to that in nontumorigenic clones of a human carcinoma cell line. Mol Cell Biol.

[b38-ehp0112-001225] Gurvich N, Klein PS (2002). Lithium and valproic acid: parallels and contrasts in diverse signaling contexts. Pharmacol Ther.

[b39-ehp0112-001225] Hancock JF (2003). Ras proteins: different signals from different locations. Nat Rev Mol Cell Biol.

[b40-ehp0112-001225] Holt D, Magos L, Webb M (1980). The interaction of cadium-induced rat renal metallothionein with bivalent mercury *in vitro*. Chem Biol Interact.

[b41-ehp0112-001225] Hughes RC (2001). Galectins as modulators of cell adhesion. Biochimie.

[b42-ehp0112-001225] Iannaccone PM (2001). Toxicogenomics: “the call of the wild chip. Environ Health Perspect.

[b43-ehp0112-001225] Jain AN, Tokuyasu TA, Snijders AM, Segraves R, Albertson DG, Pinkel D (2002). Fully automatic quantification of microarray image data. Genome Res.

[b44-ehp0112-001225] Jäkel S, Mingot JM, Schwarzmaier P, Hartmann E, Görlich D (2002). Importins fulfils a dual function as nuclear import receptors and cytoplasmic chaperones for exposed basic domains. EMBO J.

[b45-ehp0112-001225] Johannessen CU (2000). Mechanisms of action of valproate: a commentatory. Neurochem Int.

[b46-ehp0112-001225] Juriloff DM, Harris MJ (2000). Mouse models for neural tube closure defects. Hum Mol Genet.

[b47-ehp0112-001225] Kaji M, Mikawa H (1991). Induction of metallothionein in mouse liver by valproic acid. Toxicology.

[b48-ehp0112-001225] Keen CL, Peters JM, Hurley LS (1989). The effect of valproic acid on ^65^Zn distribution in the pregnant rat. J Nutr.

[b49-ehp0112-001225] Kelly DL, Rizzino A (2000). DNA microarray analyses of genes regulated during the differentiation of embryonic stem cells. Mol Reprod Dev.

[b50-ehp0112-001225] Klootwijk R, Franke B, van der Zee CE, de Boer RT, Wilms W, Hol FA (2000). A deletion encompassing *Zic3* in bent tail, a mouse model for X-linked neural tube defects. Hum Mol Genet.

[b51-ehp0112-001225] Ko MS (2001). Embryogenomics: developmental biology meets genomics. Trends Biotechnol.

[b52-ehp0112-001225] Kurisaki K, Kurisaki A, Valcourt U, Terentiev AA, Pardali K, Ten Dijke P (2003). Nuclear factor YY1 inhibits transforming growth factor beta- and bone morphogenetic protein-induced cell differentiation. Mol Cell Biol.

[b53-ehp0112-001225] Kuzuya T, Amioka K, Nabeshima T (2002). Valproic acid increases biliary copper excretion in the rat. Epilepsy Res.

[b54-ehp0112-001225] Lagger G, O’Carroll D, Rembold M, Khier H, Tischler J, Weitzer G (2002). Essential function of histone deacetylase 1 in proliferation control and CDK inhibitor repression. EMBO J.

[b55-ehp0112-001225] Lammer EJ, Sever LE, Oakley GP (1987). Teratogen update: valproic acid. Teratology.

[b56-ehp0112-001225] Lee J, Prohaska JR, Thiele DJ (2001). Essential role for mammalian copper transporter Ctr1 in copper homeostasis and embryonic development. Proc Natl Acad Sci USA.

[b57-ehp0112-001225] Lee JS, Galvin KM, Shi Y (1993). Evidence for physical interaction between the zinc-finger transcription factors YY1 and Sp1. Proc Natl Acad Sci USA.

[b58-ehp0112-001225] Liu FT, Patterson RJ, Wang JL (2002). Intracellular functions of galectins. Biochim Biophys Acta.

[b59-ehp0112-001225] Lönnstedt I, Speed TP (2002). Replicated microarray data. Statist Sinica.

[b60-ehp0112-001225] Lopez-Rovira T, Chalaux E, Massague J, Rosa JL, Ventura F (2002). Direct binding of Smad1 and Smad4 to two distinct motifs mediates bone morphogenetic protein-specific transcriptional activation of Id1 gene. J Biol Chem.

[b61-ehp0112-001225] Lu Y, Lotan R (1999). Transcriptional regulation by butyrate of mouse galectin-1 gene in embryonal carcinoma cells. Biochim Biophys Acta.

[b62-ehp0112-001225] Marks PA, Miller T, Richon VM (2003). Histone deacetylases. Curr Opin Pharmacol.

[b63-ehp0112-001225] McBurney MW (1993). P19 embryonal carcinoma cells. Int J Dev Biol.

[b64-ehp0112-001225] McMahon JA, Takada S, Zimmerman LB, Fan CM, Harland RM, McMahon AP (1998). Noggin-mediated antagonism of BMP signaling is required for growth and patterning of the neural tube and somite. Genes Dev.

[b65-ehp0112-001225] Mirkes PE (2002). 2001 Warkany lecture: to die or not to die, the role of apoptosis in normal and abnormal mammalian development. Teratology.

[b66-ehp0112-001225] Miyashita T, Yamamoto H, Nishimune Y, Nozaki M, Morita T, Matsushiro A (1994). Activation of the mouse cytokeratin A (endo A) gene in teratocarcinoma F9 cells by the histone deacetylase inhibitor Trichostatin A. FEBS Lett.

[b67-ehp0112-001225] Miyazono K Miyazawa K 2002. Id: a target of BMP signaling. Sci STKE 2002(151):PE40. 10.1126/stke.2002.151.pe40 12297674

[b68-ehp0112-001225] Moiseeva EP, Williams B, Goodall AH, Samani NJ (2003). Galectin-1 interacts with beta-1 subunit of integrin. Biochem Biophys Res Commun.

[b69-ehp0112-001225] Na L, Wartenberg M, Nau H, Hescheler J, Sauer H (2003). Anticonvulsant valproic acid inhibits cardiomyocyte differentiation of embryonic stem cells by increasing intracellular levels of reactive oxygen species. Birth Defects Res Part A Clin Mol Teratol.

[b70-ehp0112-001225] Nagai T, Aruga J, Minowa O, Sugimoto T, Ohno Y, Noda T (2000). *Zic2* regulates the kinetics of neurulation. Proc Natl Acad Sci USA.

[b71-ehp0112-001225] Nagai T, Aruga J, Takada S, Gunther T, Sporle R, Schughart K (1997). The expression of the mouse *Zic1*, *Zic2*, and *Zic3* gene suggests an essential role for Zic genes in body pattern formation. Dev Biol.

[b72-ehp0112-001225] Nau H, Hauck RS, Ehlers K (1991). Valproic acid-induced neural tube defects in mouse and human: aspects of chirality, alternative drug development, pharmacokinetics and possible mechanisms. Pharmacol Toxicol.

[b73-ehp0112-001225] Nau H, Löscher W (1986). Pharmacologic evaluation of various metabolites and analogs of valproic acid: teratogenic potencies in mice. Fundam Appl Toxicol.

[b74-ehp0112-001225] Nuwaysir EF, Bittner M, Trent J, Barrett JC, Afshari CA (1999). Microarrays and toxicology: the advent of toxicogenomics. Mol Carcinog.

[b75-ehp0112-001225] Oberemm A, Kirschbaum F (1992). Valproic acid induced abnormal development of the central nervous system of three species of amphibians: implications for neural tube defects and alternative experimental systems. Teratog Carcinog Mutagen.

[b76-ehp0112-001225] Ogra Y, Suzuki K, Gong P, Otsuka F, Koizumi S (2001). Negative regulatory role of Sp1 in metal responsive element-mediated transcriptional activation. J Biol Chem.

[b77-ehp0112-001225] Paules R (2003). Phenotypic anchoring: linking cause and effect. Environ Health Perspect.

[b78-ehp0112-001225] Perillo NL, Marcus ME, Baum LG (1998). Galectins: versatile modulators of cell adhesion, cell proliferation, and cell death. J Mol Med.

[b79-ehp0112-001225] Phiel CJ, Zhang F, Huang EY, Guenther MG, Lazar MA, Klein PS (2001). Histone deacetylase is a direct target of valproic acid, a potent anticonvulsant, mood stabilizer, and teratogen. J Biol Chem.

[b80-ehp0112-001225] Ramalho-Santos M, Yoon S, Matsuzaki Y, Mulligan RC, Melton DA (2002). “Stemness”: transcriptional profiling of embryonic and adult stem cells. Science.

[b81-ehp0112-001225] Rodriguez-Garcia MI, Morgan RO, Fernandez MR, Bances P, Fernandez MP (1999). Mouse annexin V genomic organization includes an endogenous retrovirus. Biochem J.

[b82-ehp0112-001225] Seaton BA, Dedman JR (1998). Annexins. Biometals.

[b83-ehp0112-001225] Shapland C, Hsuan JJ, Totty NF, Lawson D (1993). Purification and properties of transgelin: a transformation and shape change sensitive actin-gelling protein. J Cell Biol.

[b84-ehp0112-001225] Smith JL, Schoenwolf GC (1997). Neurulation: coming to closure. Trends Neurosci.

[b85-ehp0112-001225] Smith L, Greenfield A (2003). DNA microarrays and development. Hum Mol Genet.

[b86-ehp0112-001225] Suske G (1999). The Sp-family of transcription factors. Gene.

[b87-ehp0112-001225] Svensson K, Mattsson R, James TC, Wentzel P, Pilartz M, MacLaughlin J (1998). The paternal allele of the H19 gene is progressively silenced during early mouse development: the acetylation status of histones may be involved in the generation of variegated expression patterns. Development.

[b88-ehp0112-001225] Tanaka TS, Jaradat SA, Lim MK, Kargul GJ, Wang X, Grahovac MJ (2000). Genome-wide expression profiling of mid-gestation placenta and embryo using a 15,000 mouse developmental cDNA microarray. Proc Natl Acad Sci USA.

[b89-ehp0112-001225] Walmod PS, Skladchikova G, Kawa A, Berezin V, Bock E (1999). Antiepileptic teratogen valproic acid (VPA) modulates organisation and dynamics of the actin cytoskeleton. Cell Motil Cytoskeleton.

[b90-ehp0112-001225] Wang Y, Dooher JE, Koedood Zhao M, Gilmore TD (1999). Characterization of mouse Trip6: a putative intracellular signaling protein. Gene.

[b91-ehp0112-001225] Wegner C, Drews E, Nau H (1990). Zinc concentrations in mouse embryo and maternal plasma. Effect of valproic acid and nonteratogenic metabolite. Biol Trace Elem Res.

[b92-ehp0112-001225] Werling U, Siehler S, Litfin M, Nau H, Gottlicher M (2001). Induction of differentiation in F9 cells and activation of peroxisome proliferator-activated receptor delta by valproic acid and its teratogenic derivatives. Mol Pharmacol.

[b93-ehp0112-001225] Whitsel AI, Johnson CB, Forehand CJ (2002). An *in ovo* chicken model to study the systemic and localized teratogenic effects of valproic acid. Teratology.

[b94-ehp0112-001225] Wlodarczyk BC, Craig JC, Bennett GD, Calvin JA, Finnell RH (1996). Valproic acid-induced changes in gene expression during neurulation in a mouse model. Teratology.

[b95-ehp0112-001225] Xu W, Baribault H, Adamson ED (1998). Vinculin knockout results in heart and brain defects during embryonic development. Development.

[b96-ehp0112-001225] Yang WM, Inouye C, Zeng Y, Bearss D, Seto E (1996). Transcriptional repression by YY1 is mediated by interaction with a mammalian homolog of the yeast global regulator RPD3. Proc Natl Acad Sci USA.

[b97-ehp0112-001225] Yang YH Dudoit S Luu P Lin DM Peng V Ngai J 2002. Normalization for cDNA microarray data: a robust composite method addressing single and multiple slide systematic variation. Nucleic Acids Res 30(4):e15 [http://nar.oupjournals.org/cgi/content/full/30/4/e15]. 10.1093/nar/30.4.e15 PMC100354 11842121

[b98-ehp0112-001225] Ybot-Gonzalez P, Copp AJ (1999). Bending of the neural plate during mouse spinal neurulation is independent of actin microfilaments. Dev Dyn.

[b99-ehp0112-001225] Young MB, DiSilvestro MR, Sendera TJ, Freund J, Kriete A, Magnuson SR (2003). Analysis of gene expression in carbon tetrachloride-treated rat livers using a novel bioarray technology. Pharmacogenomics J.

[b100-ehp0112-001225] Yuan PX, Huang LD, Jiang YM, Gutkind JS, Manji HK, Chen G (2001). The mood stabilizer valproic acid activates mitogen-activated protein kinases and promotes neurite growth. J Biol Chem.

[b101-ehp0112-001225] Zimmerman AW (1984). Hyperzincemia in anencephaly and spina bifida: a clue to the pathogenesis of neural tube defects?. Neurology.

