# Supplementary material for: Valproic Acid Teratogenicity: A Toxicogenomics Approach
Source: Environ Health Perspect. 2004 Jun 3;112(12):1225–35. doi: 10.1289/txg.7034 (PMC1277116; doi:10.1289/txg.7034)
Supplement: Supplemental Tables [file ehp0112-001225s1.pdf]

Supplemental Material

Supplemental Material, Table 1. Genes responding transcriptionally to VPA in embryos

NIA EST ID	Gene Symbol[a]	Gene Name[b]	Log odds	$\log_2$ fold change	Function[c]
H3013D11	*Mt2*	metallothionein 2	11.6	1.58	Heat Shock/Stress
H3010E09	*Mt2**	metallothionein 2*	11.4	1.60	Heat Shock/Stress**
H3020C02	*Mt1*	metallothionein 1	10.3	1.26	Heat Shock/Stress
H3009D05	*Lgals*1	lectin, galactose binding, soluble 1	8.7	1.02	Matrix/Structural Proteins
H3007E10	*Ckb*	creatine kinase, brain	8.2	1.45	Matrix/Structural Proteins
H3010D09	*Vcl*	vinculin	7.0	0.79	Matrix/Structural Proteins
H3012B10	?	Mus musculus, clone MGC:6827 IMAGE:2649084, mRNA, complete cds	5.9	1.10	?**
H3054D02	*H3f3b*	H3 histone, family 3B	5.9	0.71	Transcription/Chromatin
H3013D08	*H3f3b*	H3 histone, family 3B	5.7	0.66	Transcription/Chromatin
H3042F03	*Smyd5*	SET and MYND domain containing 5	5.4	-0.64	?
H3015A10	*Pmm1*	phosphomannomutase 1	4.9	0.71	Matrix/Structural Proteins
H3030H08	*Hmgcl*	3-hydroxy-3-methylglutaryl-Coenzyme A lyase	4.8	0.62	Transcription/Chromatin
H3042E05	?	?	4.6	0.74	?
H3026G03	*Kpnb1*	karyopherin (importin) beta 1	4.5	-0.60	Matrix/Structural Proteins
H3017H12	*Tagln2*	transgelin 2	4.4	0.64	?
H3003A03	*Lgals1*	lectin, galactose binding, soluble 1	4.2	0.88	?
H3048G06	*Srm*	spermidine synthase	4.1	-0.59	?
H3058D09	*Gng3*	guanine nucleotide binding protein (G protein), gamma 3 subunit	3.5	0.56	Signal Transduction
H3028G03	*Robo1*	roundabout homolog 1 (Drosophila)	3.1	0.69	?
H3022G07	*Lgals1*	lectin, galactose binding, soluble 1	3.1	0.67	Matrix/Structural Proteins
H3032H10	?	Mus musculus xenotropic and polytropic retrovirus receptor 1, mRNA (cDNA …	3.1	0.61	?
H3024F03	*Cox7a2l*	cytochrome c oxidase subunit VIIa polypeptide 2-like	3.0	0.50	?
H3031A04	2410016F19Rik	RIKEN cDNA 2410016F19 gene	3.0	-0.74	?
H3007G03	Kpnb1	karyopherin (importin) beta 1	2.9	-0.49	Matrix/Structural Proteins
H3059F01	*Uchl1*	ubiquitin carboxy-terminal hydrolase L1	2.9	0.93	?
H3024H10	*Fignl1*	fidgetin-like 1	2.9	0.47	?
H3004B11	A930014C21Rik	RIKEN cDNA A930014C21 gene	2.8	0.51	?
H3035A06	?	?	2.8	0.70	?
H3027A06	*Eif4g2*	eukaryotic translation initiation factor 4, gamma 2	2.7	-0.48	Protein Synthesis/Translational Control
H3008A10	*Mybbp1a*	MYB binding protein (P160) 1a	2.5	-0.53	?
H3056G04	*Brd4*	bromodomain containing 4	2.5	0.76	Signal Transduction
H3014B09	*Anxa11*	annexin A11	2.2	0.58	Matrix/Structural Proteins
H3009G09	?	Mus musculus 10 days neonate cerebellum cDNA, RIKEN full-length enriched…	2.2	0.51	?
H3018F10	?	Mus musculus ES cells cDNA, RIKEN full-length enriched library, clone:C3300…	2.2	- 0.52	?

Supplemental Material, Table 1 (continued)

NIA EST ID	Gene Symbol[a]	Gene Name[b]	Log odds	Log_2 fold change	Function[c]
H3007D03	*Efs*	embryonal Fyn-associated substrate	2.1	-0.52	Signal Transduction
H3027E04	*Bcat1*	branched chain aminotransferase 1, cytosolic	2.1	-0.46	Signal Transduction
H3010E11	?	?	2.0	-0.45	?
H3064E06	A730008H23	hypothetical protein A730008H23	2.0	0.41	?
H3045F12	*Plcd*	phospholipase C, delta	2.0	0.65	Signal Transduction
H3022F11	?	?	1.9	-0.49	?
H3010H10	*Ramp2*	receptor (calcitonin) activity modifying protein 2	1.9	0.84	Signal Transduction
H3022B04	?	?	1.9	-0.45	?
H3016F11	?	?	1.9	-0.43	?*
H3040C04	*Ltb4dh*	leukotriene B4 12-hydroxydehydrogenase	1.8	0.69	?
H3031E04	*Rpo1-4*	RNA polymerase 1-4	1.8	-0.46	Protein Synthesis/Translational Control
H3003F10	*Idb1*	inhibitor of DNA binding 1	1.8	0.51	Transcription/Chromatin
H3019D11	?	?	1.7	-0.40	?
H3030C12	?	?	1.7	0.52	?
H3011D03	*Fasn*	fatty acid synthase	1.7	-0.68	Matrix/Structural Proteins
H3026H05	*D11Moh45*	DNA segment, Chr 11, KL Mohlke 45	1.6	-0.43	?
H3012D03	*Pmf1*	polyamine-modulated factor 1	1.6	-0.53	?
H3030C02	*Sdcbp*	syndecan binding protein	1.6	0.42	Matrix/Structural Proteins
H3003F11	9130023P14Rik	RIKEN cDNA 9130023P14 gene	1.6	0.63	Matrix/Structural Proteins
H3005E03	?	?	1.6	0.73	?
H3024F06	*Shmt1*	serine hydroxymethyl transferase 1 (soluble)	1.5	-0.42	Energy/Metabolism
H3008H06	?	?	1.5	0.44	?*
H3013C04	?	?	1.3	0.44	?*
H3031C09	*Trip6*	thyroid hormone receptor interactor 6	1.3	-0.45	Signal Transduction
H3010C05	1110007A10Rik	RIKEN cDNA 1110007A10 gene	1.3	0.38	?
H3017C11	*Rangnrf-pending*	RAN guanine nucleotide release factor	1.3	-0.39	Signal Transduction
H3064A03	1110046L09Rik	RIKEN cDNA 1110046L09 gene	1.3	0.45	?
H3018D02	*Gnai2*	guanine nucleotide binding protein, alpha inhibiting 2	1.2	0.40	Signal Transduction
H3001E10	*Nolc1*	nucleolar and coiled-body phosphoprotein 1	1.2	-0.46	?
H3044A03	*Crtap*	cartilage associated protein	1.2	-0.44	Matrix/Structural Proteins
H3023B11	*Hspd1*	heat shock protein 1 (chaperonin)	1.1	-0.41	Heat Shock/Stress
H3006B10	?	?	1.0	0.65	?

[a,b] Annotations are as given by the NIA web site (http://www.lgsun.grc.nia.nih.gov/cDNA/15k.html) as of May 30, 2003, except where indicated.
[b] Truncated at approximately 65 characters
[c] Annotations from SOURCE (http://source.stanford.edu/cgi-bin/source/SourceSearch) as of September 19, 2003, except where indicated.
* Annotation by tBLASTx.
** Annotation done manually.
Unknown genes and/or functions are denoted with question marks.

Supplemental Material, Table 2. Genes responding transcriptionally to VPA in embryos

NIA EST ID	Gene Symbol[a]	Gene name[b]	Leave-out number	Log_2 fold change	Function[c]
H3018G08	*Gale*	galactose-4-epimerase, UDP	E3	0.59	Energy/Metabolism
H3040E06	*Hmga2*	high mobility group AT-hook 2	E3	-0.48	?
H3017F05	*Lmna*	lamin A	E3	0.99	Matrix/Structural Proteins
H3040C04	*Ltb4dh*	leukotriene B4 12-hydroxydehydrogenase	E4	0.69	?
H3022E01	*Mbc2*	membrane bound C2 domain containing protein	E3	0.61	Energy/Metabolism
H3009G06	*Papss1*	3'-phosphoadenosine 5'-phosphosulfate synthase 1	E3	0.48	Matrix/Structural Proteins
H3050E03	*Rabl3*	RAB, member of RAS oncogene family-like 3	E3	-0.54	?
H3030A08	*Rpa1*	replication protein A1	E3	0.49	DNA Replication
H3060A04	*Rpa1*	replication protein A1	E1	0.60	DNA Replication
H3005E03	?	?	E3	0.73	?
H3007A02	?	?	E3	-0.51	?
H3016A04	?	?	E3	-0.53	?
H3022F11	?	?	E3	-0.49	?
H3060E10	?	?	E3	0,61	?

[a,b]Annotations are as given by the NIA web site (http://www.lgsun.grc.nia.nih.gov/cDNA/15k.html) as of May 30, 2003, except where indicated.
[b] Truncated at approximately 65 characters
[c] Annotations from SOURCE (http://source.stanford.edu/cgi-bin/source/SourceSearch) as of September 19, 2003, except where indicated.
Unknown genes and/or functions are denoted with question marks.

Supplemental Material, Table 3. Genes responding transcriptionally to VPA in P19 cells

NIA EST ID	Gene Symbol[a]	Gene Name[b]	Log odds	Log_2 fold change	Function[c]
H3005E03	?	?	10.6	1.29	?
H3017H12	*Tagln2*	transgelin 2	10.3	1.09	?
H3007E10	*Ckb*	creatine kinase, brain	10.0	1.01	Matrix/Structural Proteins
H3040C04	*Ltb4dh*	leukotriene B4 12-hydroxydehydrogenase	9.0	1.34	?
H3018E05	*Krt2-8*	keratin complex 2, basic, gene 8	8.6	1.01	Matrix/Structural Proteins
H3056G04	*Brd4*	bromodomain containing 4	8.6	0.81	Signal Transduction
H3054D02	*H3f3b*	H3 histone, family 3B	8.4	0.81	Transcription/Chromatin
H3001H10	*Tmsb10*	thymosin, beta 10	8.3	0.81	?
H3007G06	*Krt1-18*	keratin complex 1, acidic, gene 18	7.4	1.03	Matrix/Structural Proteins
H3003A03	*Lgals1*	lectin galactose binding, soluble 1	7.4	0.84	?
H3054A08	D830048B13	hypothetical protein D830048B13	7.3	-0.84	?
H3023C06	D10Ertd17e	DNA segment, Chr 10, ERATO Doi 17, expressed	7.1	-0.74	?
H3014B09	*Anxa11*	annexin A11	7.1	0.97	Matrix/Structural Proteins
H3013D08	*H3f3b*	H3 histone, family 3B	6.9	0.92	Transcription/Chromatin
H3026F06	9130022B02Rik	RIKEN cDNA 9130022B02 gene	6.4	-0.76	Energy/Metabolism
H3009D05	*Lgals1*	lectin, galactose binding, soluble 1	6.4	0.86	Matrix/Structural Proteins
H3027E05	*Upp*	uridine phosphorylase	6.3	-0.95	Energy/Metabolism
H3030B11	*Trh*	thyrotropin releasing hormone	6.2	0.73	?
H3061C11	D11Ertd497e	DNA segment, Chr 11, ERATO Doi 497, expressed	6.1	0.65	?
H3029H05	*Tdgf1*	teratocarcinoma-derived growth factor	5.8	0.60	Signal Transduction
H3027C11	5830446M03Rik	RIKEN cDNA 5830446M03 gene	5.8	-0.62	?
H3024F02	?	?	5.6	0.80	?
H3020F04	C130032J12Rik	RIKEN cDNA C130032J12 gene	5.5	-0.64	?
H3030F05	2310075E07Rik	RIKEN cDNA 2310075E07 gene	5.4	0.83	?
H3020C02	*Mt1*	metallothionein 1	5.4	0.71	Heat Shock/Stress
H3030B10	*Sec13l-pending*	sec13-like protein	5.3	-0.60	Matrix/Structural Proteins
H3053G02	A130038L21Rik	RIKEN cDNA A130038L21 gene	5.3	0.83	?
H3038E05	*Gdf3*	growth differentiation factor 3	5.3	-0.61	Signal Transduction
H3019G03	*Tbrg1*	transforming growth factor beta regulated gene 1	5.2	0.59	?
H3003F10	*Idb1*	inhibitor of DNA binding 1	5.2	0.65	Transcription/Chromatin
H3020C04	*Xpo4*	exportin 4	5.1	-0.66	?
H3056H05	1700007D07Rik	RIKEN cDNA 1700007D07 gene	5.1	-0.63	Energy/Metabolism
H3013C04	?	?	5.1	0.63	Heat Shock/Stress
H3004B11	A930014C21Rik	RIKEN cDNA A930014C21 gene	5.0	0.81	?

Supplemental Material, Table 3 (continued)

NIA EST ID	Gene Symbol[a]	Gene Name[b]	Log odds	Log_2 fold change	Function[c]
H3057C10	*Bpnt1*	bisphosphate 3'-nucleotidase 1	4.9	-0.62	Energy/Metabolism
H3020B10	*Nsap1-pending*	NS1-associated protein 1	4.8	-0.55	?
H3024G04	?	?	4.6	0.65	?
H3015E11	*Pdlim1*	PDZ and LIM domain 1 (elfin)	4.5	0.63	Transcription/Chromatin
H3022C12	*Igf2bp1*	insulin-like growth factor 2, binding protein 1	4.4	-0.56	?
H3048G11	*Blvrb*	biliverdin reductase B (flavin reductase (NADPH))	4.2	0.53	Energy/Metabolism
H3012D03	*Pmf1*	polyamine-modulated factor 1	4.1	-0.66	?
H3010E09	*Mt2**	metallothionein 2*	4.0	0.67	Heat Shock/Stress**
H3024F03	*Cox7a2l*	cytochrome c oxidase subunit VIIa polypeptide 2-like	4.0	0.52	?
H3005B12	*Peg10*	paternally expressed 10	3.9	0.58	?
H3029G12	?	?	3.9	-0.83	?
H3037B03	?	Mus musculus 7 days embryo whole body cDNA, RIKEN full-length enriched lib…	3.8	0.60	Transcription/Chromatin
H3039H01	*Cnn2*	calponin 2	3.8	0.74	Signal Transduction
H3011C05	?	?	3.8	0.53	?
H3019F08	*Rod1*	ROD1 regulator of differentiation 1 (S. pombe)	3.7	0.69	?
H3029D03	*Cbx5*	chromobox homolog 5 (Drosophila HP1a)	3.7	-0.60	Transcription/Chromatin
H3055H05	*Carhsp1*	calcium regulated heat stable protein 1	3.6	0.59	?
H3050H06	*Irf1*	interferon regulatory factor 1	3.6	0.76	Heat Shock/Stress
H3020A12	*Hdlbp*	high density lipoprotein (HDL) binding protein	3.6	-0.78	?
H3006A12	*Peg10*	paternally expressed 10	3.5	0.52	?
H3019G11	*Lrp10*	low-density lipoprotein receptor-related protein 10	3.5	0.60	?
H3031E04	*Rpo1-4*	RNA polymerase 1-4	3.5	-0.64	Protein Synthesis/Translational Control
H3051H09	?	?	3.5	-0.69	?
H3001C11	*Il25*	interleukin 25	3.5	0.55	?
H3059G12	*Tex292*	testis expressed gene 292	3.4	-0.55	?
H3010A08	*Tex19*	testis expressed gene 19	3.4	0.68	?
H3002C10	*Falz*	fetal Alzheimer antigen	3.3	-0.70	Transcription/Chromatin
H3048A03	?	?	3.3	-0.49	?
H3003F11	9130023P14Rik	RIKEN cDNA 9130023P14 gene	3.3	0.85	Matrix/Structural Proteins
H3038F07	*Stx3*	syntaxin 3	3.2	0.59	?
H3012F11	?	?	3.2	0.54	?
H3025B08	*Nol1*	nucleolar protein 1	3.1	-0.53	Cell Cycle
H3019A08	*Arpc1b*	actin related protein 2/3 complex, subunit 1B	3.1	0.53	Matrix/Structural Proteins
H3004H06	*Hrmt1l2*	heterogeneous nuclear ribonucleoproteins methyltransferase-like 2 (S. cerevisiae)	3.1	-0.55	Energy/Metabolism
H3022G07	*Lgals1*	lectin, galactose binding, soluble 1	3.1	0.58	Matrix/Structural Proteins
H3004C04	*Mgea5*	meningioma expressed antigen 5 (hyaluronidase)	3.0	0.55	?

Supplemental Material, Table 3 (continued)

NIA EST ID	Gene Symbol[a]	Gene Name[b]	Log odds	Log_2 fold change	Function[c]
H3013A08	*Jup*	junction plakoglobin	3.0	0.67	Matrix/Structural Proteins
H3054B08	*Inpp5f*	inositol polyphosphate-5-phosphatase F	2.9	0.69	?
H3052C02	?	?	2.9	0.54	?
H3007A03	?	?	2.8	0.60	?
H3051E08	?	?	2.8	0.52	?
H3022A06	?	?	2.8	-0.45	?
H3001B04	2010200I23Rik	RIKEN cDNA 2010200I23 gene	2.7	0.51	?
H3051E12	BC020184	cDNA sequence BC020184	2.7	0.52	?
H3028E03	9030221M09Rik	RIKEN cDNA 9030221M09 gene	2.7	0.46	Matrix/Structural Proteins
H3031A04	2410016F19Rik	RIKEN cDNA 2410016F19 gene	2.7	-0.46	?
H3009E04	2410043F08Rik	RIKEN cDNA 2410043F08 gene	2.6	0.56	?
H3027C09	3010027A04Rik	RIKEN cDNA 3010027A04 gene	2.6	-0.53	?
H3052B11	*Pctk3*	PCTAIRE-motif protein kinase 3	2.6	0.56	Signal Transduction
H3029B05	*T*	brachyury	2.6	0.82	Transcription/Chromatin
H3015D04	*Elovl6*	ELOVL family member 6, elongation of long chain fatty acids (yeast)	2.6	0.46	?
H3045A10	D11Ertd530e	DNA segment, Chr 11, ERATO Doi 530, expressed	2.6	0.68	?
H3021E06	*Epha2*	Eph receptor A2	2.6	0.59	?
H3021H05	*Pdcl*	phosducin-like	2.5	0.48	?
H3014H12	*Krt2-8*	keratin complex 2, basic, gene 8	2.5	0.65	Matrix/Structural Proteins
H3059H05	*Smp1-pending*	small membrane protein 1	2.4	0.51	Matrix/Structural Proteins
H3018H05	A430104C18Rik	RIKEN cDNA A430104C18 gene	2.4	0.56	?
H3023H11	*Ftl1*	ferritin light chain 1	2.4	0.45	Energy/Metabolism
H3027C06	*Trpm7*	transient receptor potential cation channel, subfamily M, member 7	2.3	0.48	Energy/Metabolism
H3028C07	*Gpc3*	glypican 3	2.3	0.49	Matrix/Structural Proteins
H3027A06	*Eif4g2*	eukaryotic translation initiation factor 4, gamma 2	2.3	-0.44	Protein Synthesis/Translational Control
H3007E02	*Gstp2*	glutathione S-transferase, pi 2	2.2	0.48	Energy/Metabolism
H3018F05	*Cdh3*	cadherin 3	2.2	0.45	Matrix/Structural Proteins
H3048E11	?	?	2.2	0.45	?
H3010B10	?	?	2.2	0.55	?
H3025E09	2310020L09Rik	RIKEN cDNA 2310020L09 gene	2.2	0.58	?
H3054E02	?	?	2.2	-0.56	?
H3013D11	*Mt2*	metallothionein 2	2.2	0.90	Heat Shock/Stress
H3007C05	9530090G24Rik	RIKEN cDNA 9530090G24 gene	2.1	0.51	?
H3016E04	3930401E15Rik	RIKEN cDNA 3930401E15 gene	2.0	0.48	?
H3003B04	*Tapbp*	TAP binding protein	2.0	0.51	?
H3020E06	?	?	2.0	0.47	?

Supplemental Material, Table 3 (continued9

NIA EST ID	Gene Symbol[a]	Gene Name[b]	Log odds	Log_2 fold change	Function[c]
H3031E11	*Gapd*	glyceraldehyde-3-phosphate dehydrogenase	1.9	-0.41	Energy/Metabolism
H3009E09	*Topk-pending*	T-LAK cell-originated protein kinase	1.9	0.54	?
H3047F08	?	Mus musculus 0 day neonate head cDNA, RIKEN full-length enriched library, cl…	1.9	0.45	?
H3004D04	AA408296	expressed sequence AA408296	1.9	-0.43	?
H3020A05	2600005C20Rik	RIKEN cDNA 2600005C20 gene	1.8	-0.44	?
H3011E10	*Arpc1a*	actin related protein 2/3 complex, subunit 1A	1.8	0.41	?
H3025F03	*Car14*	carbonic anhydrase 14	1.8	-0.43	?
H3023G12	?	?	1.8	0.59	?
H3048E09	6230425C22Rik	RIKEN cDNA 6230425C22 gene	1.7	-0.43	?
H3011D03	*Fasn*	fatty acid synthase	1.7	-0.46	Matrix/Structural Proteins
H3024F01	*Lrp2*	low density lipoprotein receptor-related protein 2	1.7	0.55	?
H3009H04	*Rad50*	RAD50 homolog (S. cerevisiae)	1.7	0.45	DNA Replication
H3057B11	*Tpd52l1*	tumor protein D52-like 1	1.6	0.56	Signal Transduction
H3031E10	*Ahcy*	S-adenosylhomocysteine hydrolase	1.5	-0.38	Energy/Metabolism
H3052A04	*Mor2*	malate dehydrogenase, soluble	1.5	0.49	Energy/Metabolism
H3010C05	1110007A10Rik	RIKEN cDNA 1110007A10 gene	1.5	0.41	?
H3013D07	?	?	1.4	0.42	Transcription/Chromatin
H3042C02	2310009N05Rik	RIKEN cDNA 2310009N05 gene	1.4	0.48	?
H3051H03	*Litaf*	LPS-induced TN factor	1.4	0.49	Transcription/Chromatin
H3042H07	*Smarcc1*	SWI/SNF related, matrix associated, actin dependent regulator of chromatin, sub…	1.4	-0.40	Transcription/Chromatin
H3028F03	*Ctsl*	cathepsin L	1.4	0.38	Signal Transduction
H3023B12	?	?	1.4	-0.40	Heat Shock/Stress
H3020B07	*Nap1l1*	nucleosome assembly protein 1-like 1	1.3	-0.37	Transcription/Chromatin
H3023H10	*Ftl1*	ferritin light chain 1	1.3	0.42	Energy/Metabolism
H3008A06	1200008D14Rik	RIKEN cDNA 1200008D14 gene	1.3	0.45	?
H3030E02	*Gabarapl2*	GABA(A) receptor-associated protein like 2	1.3	0.38	?
H3030E10	*Ctsb*	cathepsin B	1.2	0.41	Signal Transduction
H3014H10	*Tde1l*	tumor differentially expressed 1, like	1.2	0.38	Signal Transduction
H3060B11	1110020C13Rik	RIKEN cDNA 1110020C13 gene	1.2	0.46	?
H3059F01	*Uchl1*	ubiquitin carboxy-terminal hydrolase L1	1.2	0.59	?
H3022F11	?	?	1.2	-0.46	?
H3018B03	*Mrpl12*	mitochondrial ribosomal protein L12	1.2	-0.37	?
H3015C10	E330036L07Rik	RIKEN cDNA E330036L07 gene	1.1	-0.43	Protein Synthesis/Translational Control
H3058E07	*Mtap4*	microtubule-associated protein 4	1.1	0.43	Matrix/Structural Proteins
H3032B08	*Hmgn2*	high mobility group nucleosomal binding domain 2	1.1	-0.40	Transcription/Chromatin
H3021A05	D030051D21	hypothetical protein D030051D21	1.1	0,45	?

Supplemental Material, Table 3 (continued)

NIA EST ID	Gene Symbol[a]	Gene Name[b]	Log odds	Log_2 fold change	Function[c]
H3017E12	*Sdc3*	syndecan 3	1.1	0.47	?
H3014F04	B230312B02Rik	RIKEN cDNA B230312B02 gene	1.1	0.40	?
H3023F02	*Pip5k1b*	phosphatidylinositol-4-phosphate 5-kinase, type 1 beta	1.1	-0.43	Signal Transduction
H3010B07	5730414C17Rik	RIKEN cDNA 5730414C17 gene	1.0	0.40	?
H3027H10	*Nol5a*	nucleolar protein 5A	1.0	-0.37	?
H3013A12	*Crry*	complement receptor related protein	1.0	-0.49	Matrix/Structural Proteins
H3015H08	*Lyric-pending*	lyric	1.0	0,38	?

[a,b] Annotations are as given by the NIA web site (http://www.lgsun.grc.nia.nih.gov/cDNA/15k.html) as of May 30, 2003, except where indicated.
[b] Truncated at approximately 65 characters
[c] Annotations from SOURCE (http://source.stanford.edu/cgi-bin/source/SourceSearch) as of September 19, 2003, except where indicated.
* Annotation by tBLASTx.
** Annotation done manually.
Unknown genes and/or functions are denoted with question marks.

Supplemental Material, Table 4. Genes responding transcriptionally to VPA in P19 cells

NIA EST ID	Gene Symbol[a]	Gene name[b]	Leave-out number	Log_2 fold change	Function[c]
H3016C05	*Anxa5*	annexin A5	C4	0.94	Matrix/Structural Proteins
H3036B11	*Arhgef1*	Rho guanine nucleotide exchange factor (GEF) 1	C3	0.53	Signal Transduction
H3041D11	*Ccnd3*	cyclin D3	C3	0.64	Cell Cycle
H3033F11	*Cst3*	cystatin C	C4	0.50	?
H3013G06	E130012A19Rik	RIKEN cDNA E130012A19 gene	C3	0.69	?
H3022D03	*Galk1*	galactokinase 1	C3	0.50	Energy/Metabolism
H3003B12	*Gemin5*	gem (nuclear organelle) associated protein 5	C4	-0.71	?
H3036C12	*Hdlbp*	high density lipoprotein (HDL) binding protein	C4	-1.15	?
H3004D11	*Hic1*	hypermethylated in cancer 1	C1	0.62	?
H3030H09	*Zfp278*	zinc finger protein 278	C4	-0.54	Transcription/Chromatin
H3012E02	2310004K20Rik	RIKEN cDNA 2310004K20 gene	C3	0.74	?
H3040B01	2410016F19Rik	RIKEN cDNA 2410016F19 gene	C1	-0.75	?
H3004B10	AA408112	EST AA408112	C2	0.59	?
H3032D02	?	?	C4	-0.52	?
H3036G12	?	?	C1	0.97	?
H3060F05	?	?	C2	-0.56	Protein Synthesis/Translational Control

[a,b]Annotations are as given by the NIA web site (http://www.lgsun.grc.nia.nih.gov/cDNA/15k.html) as of May 30, 2003, except where indicated.
[b] Truncated at approximately 65 characters
[c] Annotations from SOURCE (http://source.stanford.edu/cgi-bin/source/SourceSearch) as of September 19, 2003, except where indicated.
Unknown genes and/or functions are denoted with question marks.
